# Draft Genomes of Nitrogen-fixing* Frankia* Strains Ag45/Mut15 and AgPM24 Isolated from Root Nodules of *Alnus Glutinosa*

**DOI:** 10.7150/jgen.74788

**Published:** 2022-06-06

**Authors:** Philippe Normand, Petar Pujic, Danis Abrouk, Spandana Vemulapally, Trina Guerra, Camila Carlos-Shanley, Dittmar Hahn

**Affiliations:** 1 Université Claude-Bernard Lyon 1, Université de Lyon, UMR 5557 CNRS Ecologie Microbienne, Villeurbanne, France.; 2 Texas State University, Department of Biology, 601 University Drive, San Marcos, TX 78666, USA.

**Keywords:** *Frankia*, Actinorhizal symbiosis, genome, nitrogen-fixing frankiae, biosynthetic gene clusters

## Abstract

The genomes of two nitrogen-fixing *Frankia* strains, Ag45/Mut15 and AgPM24, isolated from root nodules of *Alnus glutinosa* are described as representatives of a novel candidate species*.* Phylogenomic and ANI analyses confirmed that both strains are related to cluster 1 frankiae, and that both strains belong to a novel species. At 6.4 - 6.7 Mb, their genomes were smaller than those of other cultivated *Alnus*-infective cluster 1 strains but larger than that of the non-cultivated *Alnus*-infective cluster 1 Sp+ strain AgTrS that was their closest neighbor as assessed by ANI. Comparative genomic analyses identified genes essential for nitrogen-fixation, gene composition as regards COGs, secondary metabolites clusters and transcriptional regulators typical of those from *Alnus*-infective cluster 1 cultivated strains in both genomes. There were 459 genes present in other cultivated *Alnus*-infective strains lost in the two genomes, spread over the whole of the genome, which indicates genome erosion is taking place in these two strains.

## Introduction

The genus *Frankia* consists of nitrogen- and non-nitrogen-fixing actinobacteria that can occur in root nodules in symbiosis with a variety of woody plants 1, 2, and in soil 3. Root nodule formation is host plant-specific, with host infection groups, i.e. the *Alnus* and *Casuarina* host infection group, the Rosaceae/Coriariaceae/Datiscaceae host infection group and the Elaeagnaceae/Rhamnaceae host infection group, respectively, largely represented by *Frankia* clusters 1, 2 and 3. These clusters were established by comparative analyses of ribosomal RNA gene sequences 4 and represent nitrogen-fixing frankiae, while cluster 4 frankiae are typically unable to fix N_2_, with one exception, and are often not able to form root nodules 4, 5. Within clusters, assignment of strains to sub-clusters, OTUs, groups and genomospecies have been used to further describe diversity within the genus 4, 6-9.

Whole genome sequence analyses resulted in the description of several species within the genus *Frankia*. These analyses include isolates deposited as type strains in culture collections, as well as uncultured *Frankia* populations in root nodules of specific host plants described as candidate species 10. As summarized by Normand and Fernandez 10, cluster 1 is currently the most extensively described cluster with four species and two candidate species described, while one species and three candidate species are identified in cluster 2. Four species belong to cluster 3, and three species to cluster 4, with genomes of two additional potential species published recently 11. For cluster 1, comparative sequence analyses of amplicons of an actinobacteria-specific insertion in the 23S rRNA genes of frankiae identified several strains clustering together but distinct from type strains of cluster 1 12. These strains included strains Ag45/Mut15 and AgPM24 isolated from root nodules of *Alnus glutinosa* from two lake shores, one in Germany and one in The Netherlands about 500 km apart, i.e. Grossensee (53.631031, 10.359319) 13, and Hoogmade (52.162016, 4.591356) 7, respectively. The goal of this study was to use whole genome sequence analyses to assess the viability of our previous amplicon-based analysis, and thus affirm the potential of these strains for the description of a new species.

## Materials and Methods

### Sample preparation

*Frankia* strains Ag45/Mut15 and AgPM24 that were previously identified as members of cluster 1, representing a subcluster designated as subgroup II (14) or cluster 1b (12) were from a stock frozen at -20 °C in Defined Propionate Medium (DPM) containing propionate and NH_4_Cl as C and N source, respectively (15), at 30 °C for two weeks. Cells were harvested by centrifugation (15,000 × g, 5 min) and aggregates homogenized by brief sonication (10 s at 20% output in a S-450 sonifier, Branson Ultrasonics, Danbury, CT) (16). After an additional centrifugation, DNA was extracted from cell pellets using the SurePrep^TM^ Soil DNA Isolation Kit (Fisher Scientific, Houston, TX) (17). DNA concentrations were measured with a Qubit^®^ 2.0 Fluorometer (Life Technologies, Carlsbad, USA), and DNA sent to the Microbial Genomics Sequencing Center, Pittsburgh, PA, USA for library preparation and sequencing using the Illumina tagmentation protocol and the NextSeq Illumina platform (2 × 150 bp) using standard protocols.

### Genome assembly

Sequence reads were filtered and trimmed using the default settings of fastp (18), and reads with average %GC<54 were removed using bbduk (https://jgi.doe.gov/data-and-tools/bbtools/bb-tools-user-guide/). Genomes were assembled using SPAdes 3.13.0 19 and QUAST to check the quality of the assembled genomes 20. Their completeness was estimated using the lineage workflow (lineage_set) CheckM v1.0.18 21 with default values.

### Comparative genomic analysis

Average Nucleotide Identity (ANI) comparisons (22) were performed for all *Frankia* genomes of type strains of described species and other selected genomes using the pyani platform with the b (Blast) setting (23; https://pyani.readthedocs.io). The genomes were compared to *Frankia* genomes of type strains of isolates on the Mage platform 24 to compute clusters of orthologous genes or COGs 25, to identify secondary metabolites clusters through antiSMASH 26, and identify genes specific to the new genomes or lost in the two genomes. A phylogenetic tree was reconstructed using a MASH distance matrix 27 and the tree computed dynamically directly in the Mage browser using a rapid neighbour joining algorithm 28.

## Results

### Sequence data

CheckM analyses showed that the assembled genomes for strains Ag45/Mut15 and AgPM24 were complete with scores of 98.09 for both strains. The number of contigs was 113 and 181 for Ag45/Mut15 and AgPM24, respectively. The largest contig was 550369 and 296366 for Ag45/Mut15 and AgPM24, respectively. The strain contamination index (CheckM) was 1.09 and 0.55 for Ag45/Mut15 and AgPM24, respectively.

### Phylogenetic analysis of *Frankia* spp. Isolates

**Figure 1:**
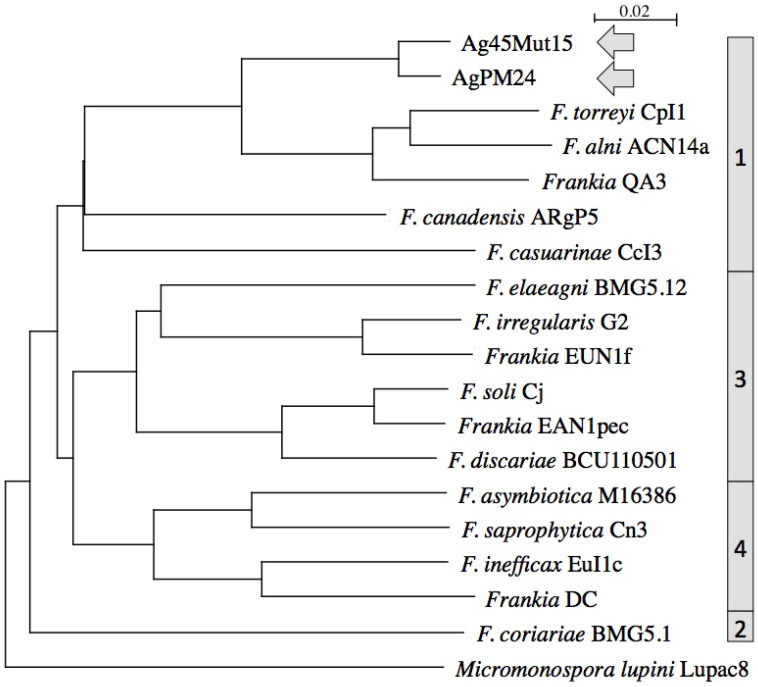
Phylogenetic tree of complete genomes using *Micromonospora lupini* (NZ_CAIE00000000.1) as outgroup. Clusters are indicated on the right.

**Figure 2:**
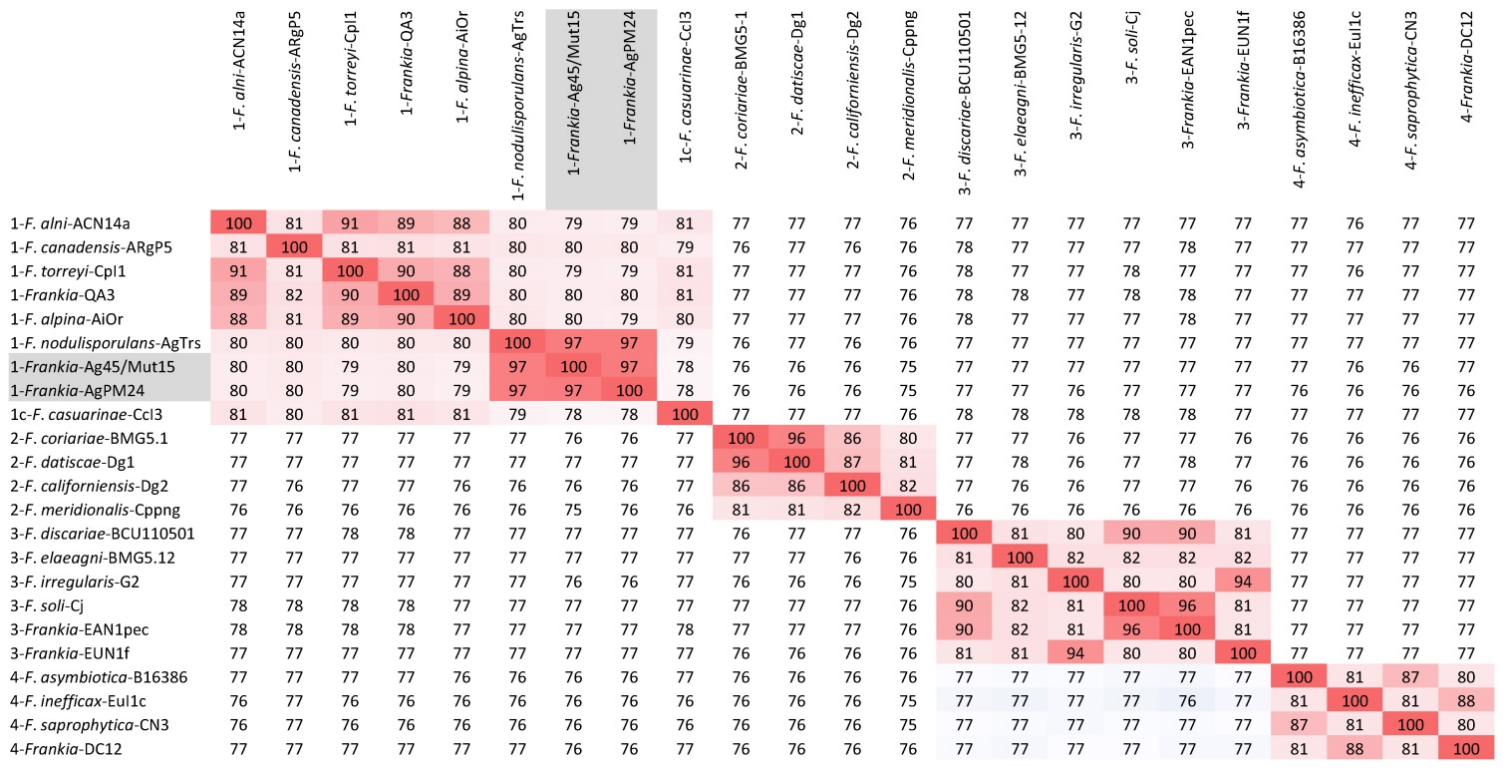
Heatmap matrix of Average Nucleotide Identity (ANI) comparisons (in percent) for the *Frankia* genomes of type strains of described species using the pyani platform with the b (Blast) setting (23); https://pyani.readthedocs.io). The two genomes described in the present study are highlighted in grey.

The two genomes were similar in size with about 6.4 Mb and 6.7 Mb, respectively, which is about 1 Mb smaller than those of other *Alnus*-infective cluster 1 cultivated strains (Table 1). They were also similar in DNA G+C% content at 71.35-71.37, which is 1% lower than values for other *Alnus*-infective cluster 1 cultivated strains. A phylogenetic tree generated from the MASH matrix with *Frankia* genomes of type strains revealed that the closest strains to Ag45/Mut15 and AgPM24 were members of cluster 1 (Figure 1). Average nucleotide identity (ANI) between strains Ag45/Mut15 and AgPM24 was 97%, indicating that they belong to a single genospecies (Figure 2). An ANI of 97% was also obtained with strain AgTrS, an uncultured *Frankia* population in root nodules representing Candidatus *Frankia nodulisporulans*. Since this strain is an obligate symbiont with a very different physiology, it will not be considered further in the present study. ANI values at or below 80% were obtained for both strains in comparison with *Frankia* genomes of type strains of all described species (Figure 2). The ANI values with other cluster 1 genomes ranged from 78% (CcI3) to 80% (ACN14a), while 75-76% values were obtained with cluster 2 genomes, and 76-77% with cluster 3 and 4 genomes (Figure 2).

### Analysis of functional genes in *Frankia* spp. isolates

Most genes associated with the *Frankia*-actinorhizal plant symbiosis were recovered in the two genomes, i.e. *nif, hup, suf, shc, cel, glx, bcsA* (Table 1). All genes that are more abundant in symbiotic lineages (clusters 1, 2 and 3) than in non-symbiotic lineages (cluster 4) (*sodF, geoA, argF, accA, rhbE, dctA, phdA*, *tgsA, ddnB*) were also recovered in Ag45/Mut15 and AgPM24 (Table 1). Conversely, *gvp* genes that code for gas vesicle proteins and one of the two *hup* clusters that are found in infective cluster 1 strains were not found in the two genomes.

The COG computation showed a set-up for Ag45/Mut15 and AgPM24 characteristic of other* Alnus*-infective cluster 1 strains with a low number of categories “N” (Cell motility) and “O” (Posttranslational modification, protein turnover, chaperones) (Table 2). These results are similar for the antiSMASH computation that showed Ag45/Mut15 and AgPM24 to have a set-up characteristic of other* Alnus*-infective cluster 1 strains with a high number of T1PKS and NRPS (Table 3) as were transcriptional regulators with a low number of GntR, IclR, LysR regulators (Table 4). A phyloprofile of genes present in Ag45/Mut15 and AgPM24 but without homologs at a threshold of 50% identity in AA and present in a synteny group in *F. alni* ACN14a, *Frankia* sp. QA3, *F. torreyi* CpI1 and *F. canadensis* ARgP5 yielded 1068 hits of which 621 were “proteins of unknown function”, 37 “HTH-transcriptional regulators”, 15 “acyl-CoA metabolism”, 5 “sigma factors”, 9 “amidohydrolase”, 14 “ABC transporter” and 7 “P450 cytochrome” (Table S1). Two were involved in the metabolism of xylose and xylulose.

A reverse study for genes present in *F. alni* ACN14a, *Frankia* sp. QA3, *F. torreyi* CpI1 and *F. canadensis* ARgP5 but absent in Ag45/Mut15 and AgPM24 yielded 459 hits among which an xanthine dehydrogenase locus, a CRISPR-locus, a acetyl/propionyl CoA carboxylase locus, an uptake hydrogenase locus, a dicarboxylate transporter, a hup locus, the GVP locus, several transporters (Table S1).

The genes lost in Ag45/Mut15 and AgPM24 have been mapped on the genome of ACN14a and found to be evenly spread over the whole genome (Supplementary Fig. 1).

## Discussion

Phylogenomic and ANI analyses confirmed that strains Ag45/Mut15 and AgPM24 are related to cluster 1 frankiae, and indicate that both strains isolated from *Alnus glutinosa* belong to a novel species. Genome sizes of both strains were about 6.4 Mb and 6.7 Mb, respectively, and thus smaller than genomes of most cluster 1 and some cluster 3 frankiae (7.5 Mb to 7.9 Mb), though genomes of *Frankia casuarinae* (4.9 to 5.6 Mb) and *F. nodulisporulans* (4.9 Mb), as well as *F. coriariae* as cluster 2 representative (5.8 Mb) were even smaller (see 5 for review). Some cluster 3 frankiae were much larger in size (9.0 to 10.4 Mb) 29-31, similar to many cluster 4 frankiae (8.8 to 10.7 Mb) 11. Frankiae with larger genomes that often result from duplications of genes involved in substrate transfers into central metabolic pathways (e.g. cluster 1, 3 and 4 frankiae), might have an enhanced potential to exploit a large variety of environments 30, 32, compared to frankiae with smaller genomes due to genome reductions that are associated to reduced saprotrophic potential, while maintaining their symbiotic potential (e.g. *F. casuarinae, F. nodulisporulans* and* F. coriariae*).

Both Ag45/Mut15 and AgPM24 belong to a group of strains within cluster 1 that are able to use leaf litter compounds as carbon resource in addition to root exudates commonly used by other strains of clusters 1, 3 and 4 16, 33. Together with cluster 3 frankiae, members of this group have been identified as major populations in soils, with absolute numbers depending on the sampling depth, physicochemical conditions and the vegetation 12, 14, 34, 35. Young stands of both host trees (e.g. natural stands of *A. glutinosa*) 35, and non-host trees (e.g. young plantations of *Betula nigra*) 14 seem to promote members of this group, as do leaf litter amendments to soils, both for introduced and indigenous populations 36. Thus, this group with strains Ag45/Mut15 and AgPM24 as representatives could be adapted to carbon resources provided by the decomposition of plant material and represent a group of frankiae characteristic of soils in early stages of plant-mediated organic matter accumulation.

Functional genes typically found in nitrogen-fixing frankiae (i.e. clusters 1, 2 and 3) within the *Frankia*-actinorhizal plant symbiosis were recovered in the genomes of strains Ag45/Mut15 and AgPM24. These strains appear to have lost a large number of genes dispensable for saprotrophic life as is the case in Sp+ lineages 37 or in cl2 lineages 32 where one megabase or more relative to the closest neighbor has been lost. This process of gene erosion is slow with seemingly a conserved possibility of growth under physiologically demanding conditions 38. The full extent of diversity with cluster 1 is slowly emerging with the description of genomes from new lineages such as the present one. It appears some lineages such as *F. torreyi* and even more so *F. casuarinae* have been isolated repeatedly while others such as QA3, *F. canadensis* and the two lineages Ag45/Mut15 and AgPM24 described in the present study have been more rarely isolated. Two related factors have probably caused this distortion, one is the evolutionary success over long eras resulting in a higher abundance in nature and the other is the physiological ability to grow relatively fast on a large range of substrates resulting in a higher isolation success rate. Their genome composition should now be analyzed in that light of adaptation to contrasted biotopes.

## Data Summary

Genomes of the strains sequenced in this study from Dr. Dittmar Hahn culture collection and were deposited in the National Center for Biotechnology Information (NCBI), under BioProject Number PRJNA680372. Whole Genome Sequencing (WGS) accession numbers are JALKFT000000000 for strain Ag45/Mut15, and JALKFW000000000 for strain AgPM24.

A list of other *Frankia* genomes utilized in this study can be found in Table 1. All sequences were downloaded from the NCBI Assembly database.

## Supplementary Material

Supplementary figure and table.

## Figures and Tables

**Table 1:** Basic genome characteristics of *Frankia* strains Ag45/Mut15 and AgPM24 compared to those of type strains of *Frankia* species in clusters 1 to 4

	Cluster 1							Cluster 2	Cluster 3						Cluster 4			
Strain	ACN14a^T^	ARgP5^T^	CpI1^T^	QA3	Ag45/Mut15	AgPM24	CcI3^T^	BMG5.1^T^	BCU110501^T^	BMG5.12^T^	G2^T^	Cj^T^	EAN1pec	EUN1f	M16386^T^	EuI1c^T^	Cn3^T^	DC12
Collection	DSM 45986	DSM 45898	DSM 44263				DSM 45818	DSM 100624	DSM 46785	DSM 46783	DSM 45899	DSM 100623			DSM 100626	DSM 45817	DSM 105290	
*Frankia* species	*alni*	*canadensis*	*torreyi*				*casuarinae*	*coriariae*	*discariae*	*elaeagni*	*irregularis*	*soli*			*asymbiotica*	*inefficax*	*saprophytica*	
Genomic G+C content (mol%)	72.8	72.4	72.4	72.6	71.37	71.35	70.1	71.0	72.3	71.7	70.9	71.1	70.94	70.82	71.93	72.3	71.8	71.93
Genome length (nt)	7497934	7730285	7624758	7590853	6443382	6672691	5433628	5795263	7891711	7589313	9537992	8061539	9035218	9322173	9435764	8815781	9978592	6884336
# CDS	6,714	7,500	7,201	7,307	6,088	6,370	5,593	6,487	7,567	6,977	8,663	8,108	9,063	9,428	8,884	8,099	9,262	6,630
# secondary metabolite clusters*	27	33	28	33	29	38	26	22	36	35	37	30	27	33	29	23	28	15
nifH**	1	1	1	1	1	1	1	1	1	1	1	1	1	1	0	0	0	0
*shc*	2	2	2	2	2	2	1	2	2	2	2	2	2	2	2	2	2	2
*hupL*	2	2	2	2	1	1	2	1	1	1	1	1	1	1	1	1	1	1
*sufD*	1	1	1	1	1	1	1	1	1	1	1	1	1	1	1	1	1	1
*celA1*	2	2	2	0	2	2	0	1	1	0	1	1	1	1	0	0	0	1
*glxA*	1	1	1	1	1	1	0	1	1	0	1	1	1	1	0	0	0	1
*bcsA*	1	1	1	0	1	1	0	1	1	0	0	1	1	1	1	1	1	1
*gvpJ*	1	1	1	0	0	0	0	0	1	1	1	1	1	1	1	1	1	1
*sodF*	1	1	1	1	1	1	1	1	1	1	1	0	1	1	0	0	0	0
*geoA*	1	1	1	1	1	1	1	1	1	1	1	1	1	0	1	1	0	0
*argG*	1	1	1	1	1	1	1	1	1	1	1	1	1	1	0	0	0	0
*accA*	1	1	1	1	1	1	1	1	1	1	1	1	1	1	1	0	0	0
*can*	2	2	2	2	2	2	2	2	2	2	2	1	2	2	0	1	0	0
*rhbE*	1	1	1	1	1	1	1	1	1	1	1	1	1	1	0	0	0	0
*lac*	1	1	1	1	1	1	1	0	1	1	1	1	1	1	0	1	1	1
*phdA*	1	1	1	1	1	1	1	1	1	1	1	1	1	1	1	0	0	0
*dctA*	1	1	1	1	1	1	1	0	1	0	1	1	1	1	0	0	0	0
*tgsA*	1	1	1	1	1	1	1	0	1	1	1	1	1	1	1	0	1	0
*ddnB*	1	1	1	1	1	1	0	0	1	1	1	1	1	1	1	0	0	0
*mopB*	1	1	2	2	2	2	1	1	2	2	2	1	2	2	0	0	0	0
*qorB*	1	2	1	1	0	0	0	0	1	1	1	1	1	1	0	0	0	0
*glbN*	1	1	1	2	1	1	1	1	1	1	1	1	1	1	0	1	0	1
# contigs	1	568	153	120	113	181	1	116	207	139	83	289	1	396	174	1	2	1
Accession	NC_008278.1	OESX01000001	JYFN00000000	WGS NZ_AJWA.1	JALKFT000000000	JALKFW000000000	CP000249.1	JWIO00000000	ARDT00000000	ARFH00000000	FAOZ00000000	MAXA00000000.1	AAII00000000	ADGX00000000	MOMC00000000	CP002299.1	AGJN00000000	LANG01000000
Reference	(30)	(39)	(40)	(41)	this study		(30)	(38)	(42)	(43)	(44)	(45)	(30)	(46)	(47)	(46)	(30)	(46)

* indicates the number of clusters identified by AntiSMASH ** indicates the number of hits (>50%) following a BlastP. *nif* is nitrogenase, *shc* is squalene hopene cyclase, *hup* is hydrogenase uptake, *suf* is sulfur-iron cluster, *cel* is cellulase, *glx* is glucose oxidase, *bcs* is cellulose synthase, *gvp* is gas vesicle cluster, *sodF* is superoxide dismutase iron, *geoA* is geosmine synthase, *arG* is arginine, *acc* is acetate carboxylase, *can* is carbonic anhydrase, *rhb* is rhizobactin, *lac* is laccase, *phd* is a phytoene desaturase, *dct* is a dicarboxylate transporter, *tgs* is diacylglycerol O-acyltransferase. *ddn* is F_420_H(2)-dependent quinone nitroreductase, *mop* is molybdenum transport, *qor* is quinone oxydoreductase, *glb* is hemoglobin.

**Table 2:** COG characteristics of *Frankia* strains Ag45/Mut15 and AgPM24 compared to those of type strains of *Frankia* species in clusters 1 to 4

Strain	Cluster 1							Cluster 2	Cluster 3						Cluster 4			
	ACN14a^T^	ARgP5^T^	CpI1^T^	QA3	Ag45/Mut15	AgPM24	CcI3^T^	BMG5.1^T^	BCU110501^T^	BMG5.12^T^	G2^T^	Cj^T^	EAN1pec	EUN1f	M16386^T^	EuI1c^T^	Cn3^T^	DC12
species	*alni*	*canadensis*	*torreyi*				*casuarinae*	*coriariae*	*discariae*	*elaeagni*	*irregularis*	*soli*			*asymbiotica*	*inefficax*	*saprophytica*	
Class^1^																		
D	56	66	75	64	56	61	57	80	65	63	80	66	65	78	63	62	64	67
M	241	189	253	236	225	241	207	203	292	259	297	248	292	311	299	258	266	255
N	19	15	26	22	12	17	12	30	20	16	28	21	20	29	16	20	11	17
O	181	134	181	190	133	140	147	149	200	165	176	200	200	195	177	173	200	149
T	325	226	320	326	291	290	232	253	405	336	436	400	405	418	415	405	494	282
U	42	38	50	38	45	50	48	50	54	53	66	56	54	64	53	52	52	50
V	94	74	86	102	77	81	60	78	107	84	117	126	107	110	130	113	153	113
J	212	226	212	257	209	212	202	243	207	197	203	219	207	226	243	241	247	232
K	565	402	594	646	509	525	369	409	739	577	778	688	739	755	785	809	945	520
L	270	254	351	356	308	319	433	289	613	398	398	518	613	468	380	286	409	399
C	435	323	455	472	346	347	256	362	492	394	527	451	492	530	555	507	589	332
E	523	386	482	534	452	451	335	396	577	461	630	516	577	623	670	661	704	447
F	111	82	104	108	96	94	94	92	107	94	103	101	107	97	129	116	114	107
G	326	274	321	342	289	297	233	249	418	326	372	360	418	428	450	426	488	302
H	192	149	186	187	170	184	174	173	187	177	192	181	187	182	188	186	208	163
I	432	258	400	460	296	303	191	297	513	412	643	405	513	619	586	624	619	313
P	311	243	323	332	307	313	210	293	381	298	408	343	381	387	402	394	427	278
Q	376	226	368	371	304	339	197	320	488	369	565	417	488	550	531	534	569	256
R	1009	704	1005	1059	814	836	619	682	1216	969	1323	1064	1216	1280	1343	1332	1508	865
S	301	226	315	286	258	278	223	243	323	297	336	328	323	338	341	334	375	284

^1^class: **D**: Cell cycle control, cell division, chromosome partitioning; **M**: Cell wall/membrane/envelope biogenesis; **N**: Cell motility; **O**: Posttranslational modification, protein turnover, chaperones; **T**: Signal transduction mechanisms; **U**: Intracellular trafficking, secretion, and vesicular transport; **V**: Defense mechanisms; **J**: Translation, ribosomal structure and biogenesis; **K**: Transcription; **L**: Replication, recombination and repair; **C**: Energy production and conversion; **E**: Amino acid transport and metabolism; **F**: Nucleotide transport and metabolism; **G**: Carbohydrate transport and metabolism; **H**: Coenzyme transport and metabolism; **I**: Lipid transport and metabolism; **P**: Inorganic ion transport and metabolism; **Q**: Secondary metabolites biosynthesis, transport and catabolism; **R**: General function prediction only; **S**: Function unknown.

**Table 3:** Number of secondary metabolites clusters (antiSMASH) of *Frankia* strains Ag45/Mut15 and AgPM24 compared to those of cultivated type strains of *Frankia* species in clusters 1 to 4

Strain	Cluster 1							Cluster 2	Cluster 3						Cluster 4			
	ACN14a^T^	ARgP5^T^	CpI1^T^	QA3	Ag45/Mut15	AgPM24	CcI3^T^	BMG5.1^T^	BCU110501^T^	BMG5.12^T^	G2^T^	Cj^T^	EAN1pec	EUN1f	M16386^T^	EuI1c^T^	Cn3^T^	DC12
species	*alni*	*canadensis*	*torreyi*				*casuarinae*	*coriariae*	*discariae*	*elaeagni*	*irregularis*	*soli*			*asymbiotica*	*inefficax*	*saprophytica*	
t1PKS^1^	6	9	8	8	9	11	1	6	16	13	6	9	5	9	6	5	2	1
t2PKS	1	3	1	3	1	1	2	2	1	1	2	1	1	2	1	2	1	1
t3PKS	1	1	1	1	1	1	0	2	0	1	1	1	1	1	3	1	1	2
otherKS	4	4	3	3	3	5	4	1	4	3	6	4	4	6	2	1	2	1
t1pks-NRPS	1	0	1	0	1	2	1	0	1	0	0	0	1	1	0	0	0	0
NRPS	3	6	2	2	6	6	0	1	1	2	9	5	3	5	4	2	7	1
terpene	5	3	5	5	4	4	4	3	4	4	3	5	4	4	5	4	4	3
lanthipeptide	1	1	1	3	0	3	6	2	4	3	2	1	4	3	1	2	1	2
bacteriocin	2	1	2	2	1	2	1	1	2	2	2	2	3	0	3	1	2	0
siderophore	1	1	1	1	1	1	1	1	1	1	1	1	1	1	0	0	0	0
lassopeptide	1	1	1	2	1	1	0	1	0	0	1	0	0	0	1	2	1	2
betalactone	0	0	0	0	0	0	0	0	0	0	2	0	0	0	1	2	0	1
thiopeptide	0	0	0	0	0	0	1	0	0	0	1	0	0	0	0	0	0	0
butyrolactone	0	0	0	0	0	0	0	0	0	0	0	0	0	0	0	0	0	0
phosphonate	1	1	0	0	0	0	0	0	0	0	0	0	0	0	0	0	0	0
arylpolyene	0	0	0	0	0	0	0	0	1	0	0	0	0	0	0	0	0	0
nucleoside	0	0	0	0	0	0	1	0	0	0	0	0	0	0	0	0	0	0
ladderane	0	0	0	0	0	0	0	1	0	0	0	0	0	0	0	0	0	0
oligosaccharide	0	0	0	0	0	0	0	0	0	0	0	0	0	0	0	0	1	0
resorcinol	0	0	0	0	0	0	0	0	0	0	0	0	0	0	0	0	1	0
LAP	0	0	0	0	0	1	0	0	0	0	0	0	0	0	0	0	0	0
other	0	2	2	3	1	0	4	1	1	5	1	1	0	1	2	1	5	1
**Total/strain**	27	33	28	33	29	38	26	22	36	35	37	30	27	33	29	23	28	15

1-tnPKS is type “n” PolyKetide Synthase, NRPS is Non Ribosomal Peptide Synthase, LAP is Linear Azole/azoline-containing Peptide.

**Table 4:** Number of transcriptional regulators of *Frankia* strains Ag45/Mut15 and AgPM24 compared to those of type strains of *Frankia* species in clusters 1 to 4

Strain	Cluster 1							Cluster 2	Cluster 3						Cluster 4			
	ACN14a^T^	ARgP5^T^	CpI1^T^	QA3	Ag45/Mut15	AgPM24	CcI3^T^	BMG5.1^T^	BCU110501^T^	BMG5.12^T^	G2^T^	Cj^T^	EAN1pec	EUN1f	M16386^T^	EuI1c^T^	Cn3^T^	DC12
species	*alni*	*canadensis*	*torreyi*				*casuarinae*	*coriariae*	*discariae*	*elaeagni*	*irregularis*	*soli*			*asymbiotica*	*inefficax*	*saprophytica*	
**Class^1^**																		
AraC	9	9	10	16	6	6	2	5	15	13	17	16	28	17	20	22	21	6
ArsR	9	6	5	1	7	6	6	5	4	4	11	6	11	9	9	16	8	8
AsnC	3	2	2	4	4	3	3	2	3	3	3	3	5	4	5	5	5	3
CRP	4	2	1	1	4	4	2	3	3	3	5	2	3	5	3	5	2	3
DeoR	4	1	0	0	1	2	0	0	2	1	0	2	4	0	2	2	2	1
DtxR	1	1	1	1	1	1	1	1	1	1	1	1	1	1	1	1	1	1
FurC	2	3	3	4	3	3	2	2	5	4	5	5	4	4	5	4	4	5
GntR	25	19	10	20	7	5	6	8	21	12	19	19	20	24	27	35	30	11
IclR	3	6	4	9	4	3	2	1	4	7	12	6	12	12	6	13	11	7
LuxR	10	19	19	36	10	14	20	15	22	9	18	15	58	40	18	64	29	14
LysR	18	16	12	22	11	10	5	5	14	10	20	13	13	17	24	20	22	13
MarR	21	19	13	33	16	15	15	23	18	20	31	25	27	30	32	33	35	19
MerR	8	17	9	22	10	10	12	4	13	12	15	13	15	17	16	19	18	7
TetR	92	78	117	127	61	65	30	47	113	77	39	126	147	156	156	191	18	59
WhiB	7	7	8	7	5	6	6	6	10	5	6	9	6	5	8	10	7	8

^1^class: **AraC**: arabinose regulator; **ArsR**: arsenic resistance; **AsnC**: asparagine synthase regulator; **CRP**: cyclic AMP receptor protein (catabolite repression); **DeoR**: deoxyribonucleoside synthesis operon regulator; **DtxR**: diphtheria toxin repressor; **FurC**: ferric uptake regulator; **GntR**: gluconate regulator; **IclR**: isocitrate lyase regulator; **LuxR**: quorum-sensing luminescence regulator; **LysR**: lysine regulator; **MarR**: Multiple antibiotic resistance regulator; **MerR**: mercury resistance regulator; **TetR**: Tetracycline repressor; **WhiB**: regulation of morphological differentiation.
