# Supplementary material for: Draft Genomes of Nitrogen-fixing Frankia Strains Ag45/Mut15 and AgPM24 Isolated from Root Nodules of Alnus Glutinosa
Source: J Genomics. 2022 Jun 6;10:49–56. doi: 10.7150/jgen.74788 (PMC9194555; doi:10.7150/jgen.74788)
Supplement: Supplementary file 1 — Supplementary figure and table. [file jgenv10p0049s1.pdf]

Supp. Table1

Genes of Frankia Ag45Mut15 with homologs in AgPm24 without homologs in ACN14a, QA3, CpI1, ARgP5

Homology constraints: minLrap ≥ 0.8 ; maxLrap ≥ 0 ; Identity ≥ 50% similarities involved in a Synteny group

1068 results

Label	Begin	End	Evidence	Gene	Product
FUMID1_v1_1000001	6414189	6414359	automatic/finished	_	protein of unknown function
FUMID1_v1_100008	2899686	2900066	automatic/finished	_	protein of unknown function
FUMID1_v1_10001	287	622	automatic/finished	_	protein of unknown function
FUMID1_v1_10002	797	1282	automatic/finished	_	Uncharacterized HTH-type transcriptional regulator Rv3095
FUMID1_v1_10003	1362	1952	automatic/finished	_	putative _PNPOx domain-containing protein
FUMID1_v1_100036	2926389	2926556	automatic/finished	_	protein of unknown function
FUMID1_v1_100048	2935523	2935963	automatic/finished	_	protein of unknown function
FUMID1_v1_100059	2951813	2952280	automatic/finished	_	SnoaL-like domain-containing protein
FUMID1_v1_10006	4200	4697	automatic/finished	_	MarR family transcriptional regulator
FUMID1_v1_100060	2952277	2953047	automatic/finished	_	putative epoxide hydrolase
FUMID1_v1_100073	2970441	2971433	automatic/finished	tauD	taurine dioxygenase
FUMID1_v1_100077	2973102	2974163	automatic/finished	_	NitT/TauT family transport system substrate-binding protein
FUMID1_v1_10008	4899	6443	automatic/finished	_	MFS transporter permease
FUMID1_v1_100084	2994003	2995262	automatic/finished	_	putative ATPase
FUMID1_v1_100085	2995259	2995876	automatic/finished	_	conserved protein of unknown function
FUMID1_v1_100099	3014392	3014730	automatic/finished	_	protein of unknown function
FUMID1_v1_10010	7413	8879	automatic/finished	_	conserved protein of unknown function
FUMID1_v1_100119	3027646	3028545	automatic/finished	_	protein of unknown function
FUMID1_v1_100128	3037329	3037949	automatic/finished	_	protein of unknown function
FUMID1_v1_100138	3048840	3049160	automatic/finished	_	protein of unknown function
FUMID1_v1_10019	15214	15417	automatic/finished	_	protein of unknown function
FUMID1_v1_10055	64706	65029	automatic/finished	_	protein of unknown function
FUMID1_v1_10056	65539	67374	automatic/finished	_	conserved exported protein of unknown function
FUMID1_v1_10058	67814	68242	automatic/finished	_	protein of unknown function
FUMID1_v1_10064	74087	75286	automatic/finished	_	Histidine kinase domain-containing protein
FUMID1_v1_10065	76153	76968	automatic/finished	_	conserved membrane protein of unknown function
FUMID1_v1_10074	85634	86713	automatic/finished	_	FAD-dependent oxidoreductase
FUMID1_v1_10078	88063	88287	automatic/finished	_	protein of unknown function
FUMID1_v1_10085	96747	97604	automatic/finished	_	TetR/AcrR family transcriptional regulator, tetracycline repressor protein
FUMID1_v1_10086	97744	99312	automatic/finished	_	peptide/nickel transport system substrate-binding protein
FUMID1_v1_10087	99309	100400	automatic/finished	_	peptide/nickel transport system permease protein
FUMID1_v1_10088	100417	101283	automatic/finished	_	peptide/nickel transport system permease protein
FUMID1_v1_10089	101280	103262	automatic/finished	_	peptide/nickel transport system ATP-binding protein
FUMID1_v1_10091	104126	104656	automatic/finished	_	membrane protein of unknown function
FUMID1_v1_10092	104692	106539	automatic/finished	_	ABC transporter related
FUMID1_v1_10093	106536	108449	automatic/finished	_	ABC transporter related
FUMID1_v1_10094	108577	109281	automatic/finished	_	TetR/AcrR family transcriptional regulator, tetracycline repressor protein
FUMID1_v1_10095	109422	109493	automatic/finished	_	protein of unknown function
FUMID1_v1_10100	113823	115091	automatic/finished	_	Diguanylate phosphodiesterase
FUMID1_v1_10101	115249	116073	automatic/finished	_	SDR family oxidoreductase
FUMID1_v1_10115	128559	130784	automatic/finished	_	eukaryotic-like serine/threonine-protein kinase
FUMID1_v1_10121	139313	139633	automatic/finished	_	protein of unknown function
FUMID1_v1_10124	142908	143060	automatic/finished	_	protein of unknown function
FUMID1_v1_10132	149280	149498	automatic/finished	_	protein of unknown function
FUMID1_v1_10148	164914	165243	automatic/finished	_	protein of unknown function
FUMID1_v1_10172	188213	189430	automatic/finished	_	conserved protein of unknown function
FUMID1_v1_10175	192140	192499	automatic/finished	_	protein of unknown function
FUMID1_v1_10176	192517	192981	automatic/finished	_	protein of unknown function
FUMID1_v1_10180	197179	197379	automatic/finished	_	protein of unknown function
FUMID1_v1_10210	230601	231878	automatic/finished	_	conserved protein of unknown function
FUMID1_v1_10214	233466	233837	automatic/finished	_	protein of unknown function
FUMID1_v1_10225	242639	243280	automatic/finished	_	protein of unknown function
FUMID1_v1_10270	285795	286265	automatic/finished	_	protein of unknown function
FUMID1_v1_10295	309721	309915	automatic/finished	_	protein of unknown function
FUMID1_v1_10299	315877	316791	automatic/finished	_	protein of unknown function
FUMID1_v1_10300	316838	319939	automatic/finished	_	conserved protein of unknown function
FUMID1_v1_10313	336206	336415	automatic/finished	_	protein of unknown function
FUMID1_v1_10316	340187	340765	automatic/finished	_	protein of unknown function
FUMID1_v1_10330	354895	357558	automatic/finished	_	ATPase involved in chromosome partitioning
FUMID1_v1_10333	359941	360174	automatic/finished	_	protein of unknown function
FUMID1_v1_10354	379031	379960	automatic/finished	_	conserved protein of unknown function
FUMID1_v1_10365	392147	392470	automatic/finished	_	protein of unknown function

FUMID1_v1_10372	398329	398733	automatic/finished	_	SEC-C motif domain protein
FUMID1_v1_10373	399052	399648	automatic/finished	_	protein of unknown function
FUMID1_v1_10375	399760	399948	automatic/finished	_	protein of unknown function
FUMID1_v1_10383	405076	407925	automatic/finished	_	Non-specific serine/threonine protein kinase
FUMID1_v1_10390	414523	414771	automatic/finished	_	protein of unknown function
FUMID1_v1_10393	416370	416612	automatic/finished	_	protein of unknown function
FUMID1_v1_10404	426135	426764	automatic/finished	inhA	Isonitrile hydratase
FUMID1_v1_10411	430108	430527	automatic/finished	_	protein of unknown function
FUMID1_v1_10415	434919	435140	automatic/finished	_	protein of unknown function
FUMID1_v1_10420	438118	439689	automatic/finished	_	Sulfatase
FUMID1_v1_10422	440741	441103	automatic/finished	_	YCII domain-containing protein
FUMID1_v1_10426	443482	443850	automatic/finished	_	protein of unknown function
FUMID1_v1_10427	444102	444233	automatic/finished	_	protein of unknown function
FUMID1_v1_10428	444296	445210	automatic/finished	_	protein of unknown function
FUMID1_v1_10429	445687	446217	automatic/finished	_	protein of unknown function
FUMID1_v1_10431	446881	448488	automatic/finished	_	MFS transporter, DHA2 family, multidrug resistance protein
FUMID1_v1_10432	448767	449783	automatic/finished	_	AraC-like DNA-binding protein
FUMID1_v1_10433	449967	450782	automatic/finished	_	Short-chain dehydrogenase/reductase
FUMID1_v1_10434	451134	451766	automatic/finished	_	DNA-binding transcriptional regulator, AcrR family
FUMID1_v1_10438	454104	455249	automatic/finished	_	Ferritin-like domain-containing protein
FUMID1_v1_10439	455575	455754	automatic/finished	_	protein of unknown function
FUMID1_v1_10440	455747	456409	automatic/finished	_	GNAT family N-acetyltransferase
FUMID1_v1_10441	456406	458166	automatic/finished	_	Histidine decarboxylase
FUMID1_v1_10442	458692	458967	automatic/finished	_	protein of unknown function
FUMID1_v1_10443	458459	458647	automatic/finished	_	protein of unknown function
FUMID1_v1_10448	462057	463844	automatic/finished	_	cellulose synthase (UDP-forming)
FUMID1_v1_10451	465248	467041	automatic/finished	yjcS	putative alkyl/aryl-sulfatase YjcS
FUMID1_v1_10452	467164	469074	automatic/finished	_	phosphoserine phosphatase RsbU/P
FUMID1_v1_10454	469829	471364	automatic/finished	_	Histidine kinase
FUMID1_v1_10457	475237	479217	automatic/finished	purL	phosphoribosylformylglycinamide synthetase
FUMID1_v1_10458	479173	480711	automatic/finished	bexJ	putative transporter
FUMID1_v1_10460	481484	482173	automatic/finished	_	TetR family transcriptional regulator
FUMID1_v1_10465	487577	487723	automatic/finished	_	protein of unknown function
FUMID1_v1_10467	489862	490581	automatic/finished	def	Peptide deformylase 1
FUMID1_v1_10487	510652	510909	automatic/finished	_	protein of unknown function
FUMID1_v1_10493	516679	517617	automatic/finished	_	Ricin-type beta-trefoil lectin domain-containing protein
FUMID1_v1_10494	517610	519310	automatic/finished	_	conserved protein of unknown function
FUMID1_v1_10495	519307	524043	automatic/finished	_	DNA segregation ATPase FtsK/SpoIIIE, S-DNA-T family
FUMID1_v1_10496	524061	524375	automatic/finished	_	WXG100 family type VII secretion target
FUMID1_v1_10497	524408	524701	automatic/finished	_	conserved protein of unknown function
FUMID1_v1_10498	524763	525776	automatic/finished	_	conserved protein of unknown function
FUMID1_v1_10499	525978	530306	automatic/finished	_	protein of unknown function
FUMID1_v1_10500	530401	534039	automatic/finished	_	protein of unknown function
FUMID1_v1_10503	537300	537935	automatic/finished	sigL	ECF RNA polymerase sigma factor SigL
FUMID1_v1_10505	539303	539773	automatic/finished	_	protein of unknown function
FUMID1_v1_110003	3098582	3098842	automatic/finished	_	protein of unknown function
FUMID1_v1_110004	3099196	3099279	automatic/finished	_	protein of unknown function
FUMID1_v1_110005	3099569	3100981	automatic/finished	_	conserved protein of unknown function
FUMID1_v1_110006	3101000	3101782	automatic/finished	_	Enoyl-CoA hydratase
FUMID1_v1_110007	3101947	3103323	automatic/finished	_	Major Facilitator Superfamily protein
FUMID1_v1_110008	3103389	3104708	automatic/finished	_	ATP-grasp domain-containing protein
FUMID1_v1_110009	3104705	3105964	automatic/finished	_	ATP-grasp domain-containing protein
FUMID1_v1_110010	3105961	3107283	automatic/finished	_	ATP-grasp domain-containing protein
FUMID1_v1_110011	3107323	3108078	automatic/finished	_	conserved protein of unknown function
FUMID1_v1_110012	3108130	3108402	automatic/finished	_	Ferredoxin
FUMID1_v1_110013	3109092	3111524	automatic/finished	_	formyl-CoA transferase
FUMID1_v1_110014	3111521	3112471	automatic/finished	_	Luciferase-like monooxygenase
FUMID1_v1_110016	3114311	3115915	automatic/finished	aknOx	Aclacinomycin-N/aclacinomycin-A oxidase
FUMID1_v1_110018	3116915	3117109	automatic/finished	_	protein of unknown function
FUMID1_v1_110019	3117259	3118581	automatic/finished	_	Branched-chain amino acid ABC transporter substrate-binding protein
FUMID1_v1_110020	3118780	3119091	automatic/finished	_	Quinol monooxygenase YgiN
FUMID1_v1_110022	3120108	3121073	automatic/finished	_	3-oxoacyl-ACP reductase
FUMID1_v1_110023	3121240	3122130	automatic/finished	_	protein of unknown function
FUMID1_v1_110025	3123085	3123951	automatic/finished	_	acyl-CoA thioesterase II
FUMID1_v1_110026	3123970	3125109	automatic/finished	_	putative epoxide hydrolase
FUMID1_v1_110033	3130492	3130575	automatic/finished	_	protein of unknown function
FUMID1_v1_110034	3130542	3131945	automatic/finished	cotB	Cyclooctat-9-en-7-ol 5-monooxygenase
FUMID1_v1_110035	3131954	3133198	automatic/finished	_	conserved protein of unknown function
FUMID1_v1_110036	3133164	3133562	automatic/finished	_	protein of unknown function

FUMID1_v1_110052	3151402	3153054	automatic/finished	_	Carboxylic ester hydrolase
FUMID1_v1_110053	3153299	3153514	automatic/finished	_	protein of unknown function
FUMID1_v1_110054	3153649	3153963	automatic/finished	_	exported protein of unknown function
FUMID1_v1_110055	3153960	3154673	automatic/finished	_	protein of unknown function
FUMID1_v1_110056	3155191	3155811	automatic/finished	_	protein of unknown function
FUMID1_v1_110057	3154670	3155326	automatic/finished	_	exported protein of unknown function
FUMID1_v1_110066	3165639	3165722	automatic/finished	_	protein of unknown function
FUMID1_v1_110067	3165728	3167020	automatic/finished	_	Branched-chain amino acid ABC transporter substrate-binding protein
FUMID1_v1_110069	3168314	3169801	automatic/finished	_	bile acid-coenzyme A ligase
FUMID1_v1_110072	3171982	3172227	automatic/finished	_	protein of unknown function
FUMID1_v1_110073	3172200	3172796	automatic/finished	_	protein of unknown function
FUMID1_v1_110075	3173544	3173810	automatic/finished	_	protein of unknown function
FUMID1_v1_110093	3188422	3189228	automatic/finished	_	O-methyltransferase
FUMID1_v1_110094	3189256	3190743	automatic/finished	_	Baeyer-Villiger monooxygenase
FUMID1_v1_110095	3190791	3191273	automatic/finished	_	conserved protein of unknown function
FUMID1_v1_110096	3191373	3192221	automatic/finished	_	TetR/AcrR family transcriptional regulator, tetracycline repressor protein
FUMID1_v1_110103	3198981	3199307	automatic/finished	_	conserved protein of unknown function
FUMID1_v1_110106	3200220	3201011	automatic/finished	_	protein of unknown function
FUMID1_v1_110110	3208239	3208856	automatic/finished	_	conserved protein of unknown function
FUMID1_v1_110111	3208929	3211916	automatic/finished	_	Transcriptional regulator, LuxR family
FUMID1_v1_110113	3213788	3214633	automatic/finished	sadH	putative oxidoreductase SadH
FUMID1_v1_110120	3219757	3219903	automatic/finished	_	protein of unknown function
FUMID1_v1_110122	3221232	3221351	automatic/finished	_	protein of unknown function
FUMID1_v1_110124	3222630	3222857	automatic/finished	_	protein of unknown function
FUMID1_v1_110139	3237821	3238630	automatic/finished	_	Short-chain dehydrogenase
FUMID1_v1_110142	3241031	3244198	automatic/finished	_	DNA helicase
FUMID1_v1_110144	3244195	3247371	automatic/finished	_	protein of unknown function
FUMID1_v1_110145	3248097	3248375	automatic/finished	_	protein of unknown function
FUMID1_v1_110147	3248375	3252085	automatic/finished	_	Helicase conserved C-terminal domain-containing protein
FUMID1_v1_110148	3252087	3254201	automatic/finished	_	conserved protein of unknown function
FUMID1_v1_110150	3255116	3255382	automatic/finished	_	protein of unknown function
FUMID1_v1_110154	3259299	3259892	automatic/finished	_	protein of unknown function
FUMID1_v1_110155	3259787	3260179	automatic/finished	_	protein of unknown function
FUMID1_v1_110156	3260469	3260612	automatic/finished	_	protein of unknown function
FUMID1_v1_110159	3261602	3262309	automatic/finished	_	protein of unknown function
FUMID1_v1_110165	3266320	3267639	automatic/finished	_	diacylglycerol O-acyltransferase / wax synthase
FUMID1_v1_110167	3268072	3268725	automatic/finished	_	exported protein of unknown function
FUMID1_v1_110169	3270025	3271080	automatic/finished	_	4HBT domain-containing protein
FUMID1_v1_110170	3270548	3271237	automatic/finished	_	protein of unknown function
FUMID1_v1_110176	3276886	3277380	automatic/finished	_	NUDIX hydrolase
FUMID1_v1_110178	3277736	3278245	automatic/finished	_	protein of unknown function
FUMID1_v1_1110001	6424065	6424658	automatic/finished	_	protein of unknown function
FUMID1_v1_1120003	6425236	6425367	automatic/finished	_	protein of unknown function
FUMID1_v1_1130001	6425488	6425982	automatic/finished	_	protein of unknown function
FUMID1_v1_1140001	6426077	6426457	automatic/finished	_	protein of unknown function
FUMID1_v1_1140002	6426504	6426581	automatic/finished	_	protein of unknown function
FUMID1_v1_1170001	6428012	6428365	automatic/finished	_	protein of unknown function
FUMID1_v1_120008	3293521	3293649	automatic/finished	_	protein of unknown function
FUMID1_v1_120009	3293784	3294353	automatic/finished	_	protein of unknown function
FUMID1_v1_120010	3293966	3294730	automatic/finished	_	lysozyme
FUMID1_v1_120022	3308971	3309186	automatic/finished	_	protein of unknown function
FUMID1_v1_120031	3318173	3318370	automatic/finished	_	conserved protein of unknown function
FUMID1_v1_120070	3363627	3365147	automatic/finished	_	Protein kinase domain-containing protein
FUMID1_v1_120076	3370394	3370636	automatic/finished	_	protein of unknown function
FUMID1_v1_120077	3370728	3370997	automatic/finished	_	protein of unknown function
FUMID1_v1_120078	3370909	3371337	automatic/finished	_	protein of unknown function
FUMID1_v1_120080	3371274	3371984	automatic/finished	_	protein of unknown function
FUMID1_v1_120084	3374094	3376409	automatic/finished	_	conserved protein of unknown function
FUMID1_v1_120085	3376418	3377284	automatic/finished	_	protein of unknown function
FUMID1_v1_120086	3377247	3377753	automatic/finished	_	protein of unknown function
FUMID1_v1_120087	3377605	3377853	automatic/finished	_	protein of unknown function
FUMID1_v1_120096	3384250	3385866	automatic/finished	_	MFS transporter
FUMID1_v1_120104	3393661	3395544	automatic/finished	_	conserved protein of unknown function
FUMID1_v1_120105	3395545	3396474	automatic/finished	_	eukaryotic-like serine/threonine-protein kinase
FUMID1_v1_120109	3399539	3399784	automatic/finished	_	conserved protein of unknown function
FUMID1_v1_120111	3400525	3401442	automatic/finished	_	conserved protein of unknown function
FUMID1_v1_120113	3402554	3402997	automatic/finished	_	conserved protein of unknown function
FUMID1_v1_120115	3403015	3403215	automatic/finished	_	protein of unknown function
FUMID1_v1_120124	3413395	3414138	automatic/finished	_	protein of unknown function

FUMID1_v1_120128	3417626	3417715	automatic/finished	_	protein of unknown function
FUMID1_v1_120140	3428705	3429823	automatic/finished	_	conserved protein of unknown function
FUMID1_v1_120143	3432263	3432508	automatic/finished	_	protein of unknown function
FUMID1_v1_120158	3447931	3448452	automatic/finished	_	protein of unknown function
FUMID1_v1_130007	3457090	3457995	automatic/finished	_	MDMPI_N domain-containing protein
FUMID1_v1_130008	3457985	3458830	automatic/finished	_	Short-chain dehydrogenase
FUMID1_v1_130010	3458945	3459646	automatic/finished	_	periplasmic copper chaperone A
FUMID1_v1_130012	3461869	3461985	automatic/finished	_	protein of unknown function
FUMID1_v1_130014	3462087	3462827	automatic/finished	_	conserved protein of unknown function
FUMID1_v1_130016	3463198	3464037	automatic/finished	_	Acyl-CoA dehydrogenase
FUMID1_v1_130017	3464041	3465129	automatic/finished	_	Alkylation response protein AidB-like acyl-CoA dehydrogenase
FUMID1_v1_130020	3466600	3466674	automatic/finished	_	protein of unknown function
FUMID1_v1_130021	3466696	3467625	automatic/finished	_	conserved membrane protein of unknown function
FUMID1_v1_130022	3467829	3468236	automatic/finished	_	protein of unknown function
FUMID1_v1_130024	3470380	3471477	automatic/finished	_	Phosphotransferase enzyme family protein
FUMID1_v1_130029	3476053	3476898	automatic/finished	_	protein of unknown function
FUMID1_v1_130033	3482023	3482955	automatic/finished	_	putative membrane protein
FUMID1_v1_130034	3483999	3485231	automatic/finished	_	benzoate membrane transport protein
FUMID1_v1_130038	3486405	3487223	automatic/finished	_	conserved protein of unknown function
FUMID1_v1_130039	3487266	3488198	automatic/finished	_	protein of unknown function
FUMID1_v1_130045	3493182	3493373	automatic/finished	_	protein of unknown function
FUMID1_v1_130053	3497189	3498445	automatic/finished	_	Septum_form domain-containing protein
FUMID1_v1_130054	3498646	3498948	automatic/finished	_	protein of unknown function
FUMID1_v1_130057	3499648	3499797	automatic/finished	_	protein of unknown function
FUMID1_v1_130058	3499809	3500564	automatic/finished	_	conserved protein of unknown function
FUMID1_v1_130059	3500567	3500878	automatic/finished	_	conserved protein of unknown function
FUMID1_v1_130060	3500869	3500979	automatic/finished	_	protein of unknown function
FUMID1_v1_130061	3500957	3501796	automatic/finished	_	protein of unknown function
FUMID1_v1_130115	3551129	3551515	automatic/finished	_	protein of unknown function
FUMID1_v1_130118	3553010	3553204	automatic/finished	_	protein of unknown function
FUMID1_v1_130128	3561877	3561969	automatic/finished	_	protein of unknown function
FUMID1_v1_130139	3570715	3571218	automatic/finished	_	Nuclear transport factor 2 family protein
FUMID1_v1_130142	3572819	3573715	automatic/finished	_	Esterase
FUMID1_v1_130143	3573858	3574301	automatic/finished	cynS	Cyanate hydratase
FUMID1_v1_130147	3577512	3577898	automatic/finished	_	conserved exported protein of unknown function
FUMID1_v1_130154	3583530	3586181	automatic/finished	yppS	putative PEP-dependent enzyme
FUMID1_v1_130155	3586178	3586699	automatic/finished	_	conserved protein of unknown function
FUMID1_v1_130158	3588251	3589252	automatic/finished	_	conserved exported protein of unknown function
FUMID1_v1_130159	3589026	3589385	automatic/finished	_	protein of unknown function
FUMID1_v1_130160	3589410	3589847	automatic/finished	_	DNA-binding transcriptional regulator, MarR family
FUMID1_v1_130161	3589856	3591499	automatic/finished	_	conserved protein of unknown function
FUMID1_v1_130162	3591255	3591629	automatic/finished	_	protein of unknown function
FUMID1_v1_1360001	6435986	6436252	automatic/finished	_	protein of unknown function
FUMID1_v1_1390001	6437114	6437329	automatic/finished	_	protein of unknown function
FUMID1_v1_140014	3608298	3609290	automatic/finished	_	conserved exported protein of unknown function
FUMID1_v1_140019	3613046	3615457	automatic/finished	_	putative drug exporter of the RND superfamily
FUMID1_v1_140021	3616308	3617207	automatic/finished	_	conserved membrane protein of unknown function
FUMID1_v1_140027	3621825	3622619	automatic/finished	_	Predicted DNA-binding transcriptional regulator YafY, contains an HTH and WYL domains
FUMID1_v1_140029	3622816	3623196	automatic/finished	_	VOC family protein
FUMID1_v1_140043	3640708	3641091	automatic/finished	_	protein of unknown function
FUMID1_v1_140046	3642750	3643934	automatic/finished	_	conserved protein of unknown function
FUMID1_v1_140047	3643931	3645133	automatic/finished	_	Cytochrome P450
FUMID1_v1_140048	3645141	3646301	automatic/finished	_	Acyl-CoA dehydrogenase
FUMID1_v1_140049	3646370	3647209	automatic/finished	_	Short-chain dehydrogenase
FUMID1_v1_140051	3647903	3648739	automatic/finished	_	exported protein of unknown function
FUMID1_v1_140054	3651646	3652572	automatic/finished	_	Luciferase-like monooxygenase
FUMID1_v1_140055	3652710	3653054	automatic/finished	_	membrane protein of unknown function
FUMID1_v1_140056	3653348	3653935	automatic/finished	_	Regulatory protein TetR
FUMID1_v1_140058	3654752	3654895	automatic/finished	_	protein of unknown function
FUMID1_v1_140077	3673740	3675068	automatic/finished	_	Branched-chain amino acid ABC transporter substrate-binding protein
FUMID1_v1_140078	3675150	3676508	automatic/finished	_	Carnitine dehydratase
FUMID1_v1_140079	3676505	3677866	automatic/finished	_	benzylsuccinate CoA-transferase BbsF subunit
FUMID1_v1_140080	3678070	3679329	automatic/finished	_	Amidohydrolase
FUMID1_v1_140086	3682173	3683861	automatic/finished	mig	Medium-chain acyl-CoA ligase Mig
FUMID1_v1_140087	3683937	3684902	automatic/finished	_	LLM class F420-dependent oxidoreductase
FUMID1_v1_140088	3684899	3685885	automatic/finished	_	protein of unknown function
FUMID1_v1_140095	3690340	3691050	automatic/finished	_	Alkyl hydroperoxide reductase
FUMID1_v1_140106	3703387	3704169	automatic/finished	_	protein-tyrosine phosphatase
FUMID1_v1_140108	3705582	3706067	automatic/finished	_	conserved protein of unknown function

FUMID1_v1_140113	3707406	3708245	automatic/finished	_	3-oxoacyl-ACP reductase
FUMID1_v1_140114	3708316	3709470	automatic/finished	_	flavin-dependent trigonelline monooxygenase, oxygenase component
FUMID1_v1_140115	3709474	3709725	automatic/finished	_	protein of unknown function
FUMID1_v1_140116	3709778	3710704	automatic/finished	_	conserved protein of unknown function
FUMID1_v1_140117	3711063	3711917	automatic/finished	_	epsilon-lactone hydrolase
FUMID1_v1_140118	3712226	3712489	automatic/finished	_	protein of unknown function
FUMID1_v1_140125	3718688	3719254	automatic/finished	_	protein of unknown function
FUMID1_v1_140126	3719797	3720927	automatic/finished	adhB	Alcohol dehydrogenase B
FUMID1_v1_140129	3726050	3727297	automatic/finished	_	Branched-chain amino acid ABC transporter substrate-binding protein
FUMID1_v1_140130	3726390	3727310	automatic/finished	_	protein of unknown function
FUMID1_v1_140133	3727972	3729216	automatic/finished	_	Branched-chain amino acid ABC transporter substrate-binding protein
FUMID1_v1_140134	3729768	3730433	automatic/finished	_	Transcriptional regulator, TetR family
FUMID1_v1_140135	3730490	3730933	automatic/finished	_	DUF393 domain-containing protein
FUMID1_v1_140137	3732436	3734418	automatic/finished	_	Major facilitator superfamily MFS_1
FUMID1_v1_140139	3735436	3736524	automatic/finished	_	dihydroflavonol-4-reductase
FUMID1_v1_150006	3745199	3745408	automatic/finished	_	protein of unknown function
FUMID1_v1_150011	3749699	3749971	automatic/finished	_	conserved protein of unknown function
FUMID1_v1_150012	3750215	3750364	automatic/finished	_	protein of unknown function
FUMID1_v1_150016	3751917	3752108	automatic/finished	_	protein of unknown function
FUMID1_v1_150020	3754710	3756977	automatic/finished	_	protein of unknown function
FUMID1_v1_150022	3758854	3759243	automatic/finished	_	protein of unknown function
FUMID1_v1_150028	3763968	3764303	automatic/finished	_	protein of unknown function
FUMID1_v1_150032	3765142	3766143	automatic/finished	_	protein of unknown function
FUMID1_v1_150040	3772722	3773000	automatic/finished	_	protein of unknown function
FUMID1_v1_150052	3786662	3786904	automatic/finished	_	protein of unknown function
FUMID1_v1_150076	3809544	3810722	automatic/finished	_	protein of unknown function
FUMID1_v1_150087	3822027	3822551	automatic/finished	_	protein of unknown function
FUMID1_v1_150102	3837697	3838446	automatic/finished	_	exported protein of unknown function
FUMID1_v1_150103	3838199	3838675	automatic/finished	_	protein of unknown function
FUMID1_v1_150116	3854299	3854511	automatic/finished	_	protein of unknown function
FUMID1_v1_150121	3859869	3860861	automatic/finished	_	protein of unknown function
FUMID1_v1_150139	3880738	3880950	automatic/finished	_	protein of unknown function
FUMID1_v1_150140	3881108	3881332	automatic/finished	_	conserved protein of unknown function
FUMID1_v1_150141	3881733	3882200	automatic/finished	_	protein of unknown function
FUMID1_v1_150142	3882216	3884654	automatic/finished	_	AAA_16 domain-containing protein
FUMID1_v1_160015	3897958	3898581	automatic/finished	_	conserved protein of unknown function
FUMID1_v1_160025	3909080	3909472	automatic/finished	_	conserved membrane protein of unknown function
FUMID1_v1_160028	3912045	3912971	automatic/finished	_	conserved protein of unknown function
FUMID1_v1_160029	3913292	3913792	automatic/finished	_	protein of unknown function
FUMID1_v1_160044	3928378	3929022	automatic/finished	_	MDMPI_N domain-containing protein
FUMID1_v1_160045	3929200	3929811	automatic/finished	_	Calcium-binding protein
FUMID1_v1_160046	3929019	3929198	automatic/finished	_	protein of unknown function
FUMID1_v1_160052	3931634	3932464	automatic/finished	_	protein of unknown function
FUMID1_v1_160054	3932494	3933096	automatic/finished	_	conserved membrane protein of unknown function
FUMID1_v1_160061	3939175	3939876	automatic/finished	_	membrane protein of unknown function
FUMID1_v1_160062	3940456	3941319	automatic/finished	_	Hydrolase, alpha/beta fold family
FUMID1_v1_160068	3943920	3944492	automatic/finished	_	conserved protein of unknown function
FUMID1_v1_160069	3944627	3945481	automatic/finished	_	Gluconolactonase
FUMID1_v1_160073	3949298	3950134	automatic/finished	_	Methyltransf_25 domain-containing protein
FUMID1_v1_160074	3950131	3951036	automatic/finished	vgb	Virginiamycin B lyase
FUMID1_v1_160087	3962622	3963428	automatic/finished	_	Enoyl-CoA hydratase/isomerase
FUMID1_v1_160091	3965999	3966454	automatic/finished	_	Cupin 2 conserved barrel domain protein
FUMID1_v1_160111	3981553	3982119	automatic/finished	_	conserved membrane protein of unknown function
FUMID1_v1_160119	3987760	3988431	automatic/finished	lutC	Lactate utilization protein C
FUMID1_v1_160145	4015589	4015792	automatic/finished	_	protein of unknown function
FUMID1_v1_170001	4017222	4018346	automatic/finished	_	protein of unknown function
FUMID1_v1_170003	4019410	4020261	automatic/finished	_	Alpha/beta hydrolase
FUMID1_v1_170004	4020304	4020957	automatic/finished	_	TetR/AcrR family transcriptional regulator
FUMID1_v1_170016	4031567	4031989	automatic/finished	_	protein of unknown function
FUMID1_v1_170021	4034985	4035485	automatic/finished	_	conserved membrane protein of unknown function
FUMID1_v1_170026	4040517	4040699	automatic/finished	_	protein of unknown function
FUMID1_v1_170035	4054970	4057609	automatic/finished	_	eukaryotic-like serine/threonine-protein kinase
FUMID1_v1_170068	4093540	4094286	automatic/finished	_	N-acetyltransferase
FUMID1_v1_170076	4102296	4103513	automatic/finished	_	protein of unknown function
FUMID1_v1_170086	4114272	4115414	automatic/finished	_	conserved membrane protein of unknown function
FUMID1_v1_170088	4116643	4116870	automatic/finished	_	protein of unknown function
FUMID1_v1_170095	4125157	4125507	automatic/finished	_	protein of unknown function
FUMID1_v1_170105	4138047	4138127	automatic/finished	_	protein of unknown function
FUMID1_v1_170111	4142009	4142605	automatic/finished	_	Bifunctional deaminase-reductase domain protein

FUMID1_v1_170112	4142637	4143455	automatic/finished	_	conserved protein of unknown function
FUMID1_v1_170113	4143655	4144029	automatic/finished	_	conserved protein of unknown function
FUMID1_v1_170114	4144175	4144597	automatic/finished	_	conserved membrane protein of unknown function
FUMID1_v1_170118	4145720	4145920	automatic/finished	_	protein of unknown function
FUMID1_v1_170120	4147033	4147788	automatic/finished	_	conserved exported protein of unknown function
FUMID1_v1_170121	4147806	4148015	automatic/finished	_	protein of unknown function
FUMID1_v1_180002	4150036	4151328	automatic/finished	_	conserved protein of unknown function
FUMID1_v1_180007	4155409	4155996	automatic/finished	_	protein of unknown function
FUMID1_v1_180018	4165562	4167061	automatic/finished	_	conserved protein of unknown function
FUMID1_v1_180030	4175697	4176920	automatic/finished	_	Amidohydrolase 2
FUMID1_v1_180037	4184798	4186234	automatic/finished	_	Repeat domain-containing protein
FUMID1_v1_180070	4224535	4224858	automatic/finished	_	exported protein of unknown function
FUMID1_v1_180075	4229529	4230737	automatic/finished	_	Predicted metal-dependent hydrolase, TIM-barrel fold
FUMID1_v1_180077	4232347	4233210	automatic/finished	_	Alpha/beta fold hydrolase
FUMID1_v1_180084	4240360	4240863	automatic/finished	_	conserved protein of unknown function
FUMID1_v1_180085	4240900	4244211	automatic/finished	_	Tetratricopeptide repeat/TIR domain-containing protein
FUMID1_v1_180090	4251300	4252217	automatic/finished	_	Short-chain dehydrogenase
FUMID1_v1_180091	4252340	4253113	automatic/finished	_	putative EthD domain-containing protein
FUMID1_v1_180092	4253168	4253836	automatic/finished	_	TetR/AcrR family transcriptional regulator
FUMID1_v1_180094	4256163	4257083	automatic/finished	_	conserved protein of unknown function
FUMID1_v1_180095	4257191	4257850	automatic/finished	_	TetR/AcrR family transcriptional regulator
FUMID1_v1_180102	4266647	4266874	automatic/finished	_	protein of unknown function
FUMID1_v1_190001	4274076	4274651	automatic/finished	_	protein of unknown function
FUMID1_v1_190021	4299197	4302073	automatic/finished	_	Signal transduction histidine kinase
FUMID1_v1_190046	4345440	4345661	automatic/finished	_	protein of unknown function
FUMID1_v1_190055	4353983	4354177	automatic/finished	_	protein of unknown function
FUMID1_v1_190056	4354165	4356057	automatic/finished	_	conserved protein of unknown function
FUMID1_v1_190072	4366052	4366432	automatic/finished	_	protein of unknown function
FUMID1_v1_190073	4366770	4367009	automatic/finished	_	protein of unknown function
FUMID1_v1_190082	4373152	4373385	automatic/finished	_	protein of unknown function
FUMID1_v1_190086	4374764	4376167	automatic/finished	_	nicotinate phosphoribosyltransferase
FUMID1_v1_200007	4387786	4388022	automatic/finished	_	protein of unknown function
FUMID1_v1_200008	4387994	4388398	automatic/finished	_	protein of unknown function
FUMID1_v1_200013	4393990	4394262	automatic/finished	_	protein of unknown function
FUMID1_v1_20002	551881	552825	automatic/finished	_	LysR family transcriptional regulator, nod-box dependent transcriptional activator
FUMID1_v1_200024	4407511	4407906	automatic/finished	_	protein of unknown function
FUMID1_v1_200029	4410630	4410968	automatic/finished	_	protein of unknown function
FUMID1_v1_20003	553043	554323	automatic/finished	xyoA	putative xylitol oxidase
FUMID1_v1_200033	4414716	4415255	automatic/finished	_	conserved protein of unknown function
FUMID1_v1_200038	4420712	4420894	automatic/finished	_	protein of unknown function
FUMID1_v1_200059	4442697	4443599	automatic/finished	_	Helix-turn-helix domain-containing protein
FUMID1_v1_200060	4443909	4445564	automatic/finished	_	AMP-dependent synthetase
FUMID1_v1_200061	4445681	4446748	automatic/finished	_	Aminoglycoside phosphotransferase
FUMID1_v1_200062	4447244	4448452	automatic/finished	_	CoA transferase
FUMID1_v1_200063	4448449	4449003	automatic/finished	_	conserved protein of unknown function
FUMID1_v1_200065	4449000	4450205	automatic/finished	_	Thiolase family protein
FUMID1_v1_200066	4450300	4451769	automatic/finished	_	Aldehyde dehydrogenase family protein
FUMID1_v1_200067	4451873	4453093	automatic/finished	_	Amidohydrolase
FUMID1_v1_200068	4453098	4453355	automatic/finished	_	ferredoxin
FUMID1_v1_200069	4453547	4454785	automatic/finished	_	Cytochrome P450
FUMID1_v1_200070	4454822	4455265	automatic/finished	ddn	Deazaflavin-dependent nitroreductase
FUMID1_v1_200071	4455467	4456216	automatic/finished	_	protein of unknown function
FUMID1_v1_200072	4456277	4457143	automatic/finished	_	Flavin-dependent oxidoreductase, F420-dependent methylene-tetrahydromethanopterin reductase
FUMID1_v1_200074	4458908	4459339	automatic/finished	_	conserved protein of unknown function
FUMID1_v1_200083	4473915	4474211	automatic/finished	_	protein of unknown function
FUMID1_v1_200086	4475575	4479357	automatic/finished	_	AAA_27 domain-containing protein
FUMID1_v1_200087	4479359	4480588	automatic/finished	_	DNA repair protein SbcD/Mre11
FUMID1_v1_200089	4481202	4482263	automatic/finished	_	EndoU_bacteria domain-containing protein
FUMID1_v1_20010	559172	560059	automatic/finished	_	Universal stress protein UspA
FUMID1_v1_20012	560756	561598	automatic/finished	_	protein of unknown function
FUMID1_v1_20014	561627	562526	automatic/finished	_	Zn-dependent protease with chaperone function
FUMID1_v1_20018	567223	567606	automatic/finished	_	protein of unknown function
FUMID1_v1_20019	567585	568223	automatic/finished	_	CBS domain-containing protein
FUMID1_v1_20021	569538	569714	automatic/finished	_	protein of unknown function
FUMID1_v1_20025	572496	572909	automatic/finished	_	DUF4440 domain-containing protein
FUMID1_v1_20026	573092	573583	automatic/finished	_	DNA-binding transcriptional repressor MarR
FUMID1_v1_20027	573666	574202	automatic/finished	_	SnoaL-like domain protein
FUMID1_v1_20031	577067	577240	automatic/finished	_	protein of unknown function
FUMID1_v1_20054	597307	598050	automatic/finished	_	conserved protein of unknown function

FUMID1_v1_20059	601057	602604	automatic/finished	_	Major facilitator superfamily MFS_1
FUMID1_v1_20066	611483	612628	automatic/finished	_	Clp protease
FUMID1_v1_20068	613062	614213	automatic/finished	_	Winged helix DNA-binding domain-containing protein
FUMID1_v1_20075	624717	625934	automatic/finished	_	Peripla_BP_6 domain-containing protein
FUMID1_v1_20077	626244	627149	automatic/finished	ucpA	Oxidoreductase UcpA
FUMID1_v1_20079	628297	628959	automatic/finished	_	Regulatory protein TetR
FUMID1_v1_20080	629093	630337	automatic/finished	cyp	Methyl-branched lipid omega-hydroxylase
FUMID1_v1_20081	630601	632364	automatic/finished	_	Diguanylate cyclase (GGDEF) domain-containing protein
FUMID1_v1_20102	656428	656820	automatic/finished	_	serine/threonine-protein kinase RsbW
FUMID1_v1_20118	670019	670213	automatic/finished	_	protein of unknown function
FUMID1_v1_20164	706945	707859	automatic/finished	_	protein of unknown function
FUMID1_v1_20168	711790	711918	automatic/finished	_	protein of unknown function
FUMID1_v1_20171	714700	714891	automatic/finished	_	protein of unknown function
FUMID1_v1_20178	720991	721482	automatic/finished	_	protein of unknown function
FUMID1_v1_20199	744799	745203	automatic/finished	_	protein of unknown function
FUMID1_v1_20253	797834	799156	automatic/finished	_	conserved protein of unknown function
FUMID1_v1_20261	807845	808111	automatic/finished	_	protein of unknown function
FUMID1_v1_20317	851117	852805	automatic/finished	_	conserved protein of unknown function
FUMID1_v1_20345	877471	878883	automatic/finished	_	conserved protein of unknown function
FUMID1_v1_20355	889052	889252	automatic/finished	_	protein of unknown function
FUMID1_v1_20357	891099	891386	automatic/finished	_	protein of unknown function
FUMID1_v1_210003	4484181	4484834	automatic/finished	_	protein of unknown function
FUMID1_v1_210013	4495856	4496743	automatic/finished	_	Alpha/beta hydrolase
FUMID1_v1_210025	4509819	4510541	automatic/finished	_	carboxymethylenebutenolidase
FUMID1_v1_210027	4510800	4511216	automatic/finished	_	protein of unknown function
FUMID1_v1_210059	4547811	4548278	automatic/finished	_	protein of unknown function
FUMID1_v1_210060	4548085	4548348	automatic/finished	_	protein of unknown function
FUMID1_v1_210074	4558734	4558928	automatic/finished	_	protein of unknown function
FUMID1_v1_210086	4571032	4571208	automatic/finished	_	protein of unknown function
FUMID1_v1_210093	4577280	4578146	automatic/finished	_	conserved protein of unknown function
FUMID1_v1_220015	4593298	4594236	automatic/finished	_	TauD domain-containing protein
FUMID1_v1_220017	4595547	4596524	automatic/finished	_	ornithine carbamoyltransferase
FUMID1_v1_230002	4665682	4666653	automatic/finished	_	LysR family transcriptional regulator, nod-box dependent transcriptional activator
FUMID1_v1_230003	4666742	4667761	automatic/finished	_	aminocarboxymuconate-semialdehyde decarboxylase
FUMID1_v1_230006	4669981	4671324	automatic/finished	_	Branched-chain amino acid ABC transporter substrate-binding protein
FUMID1_v1_230010	4673057	4674028	automatic/finished	_	Kynurenine formamidase
FUMID1_v1_230016	4677285	4677422	automatic/finished	_	protein of unknown function
FUMID1_v1_230018	4678577	4680211	automatic/finished	fadD	3-[(3aS,4S,7aS)-7a-methyl-1,5-dioxo-octahydro-1H-inden-4-yl]propanoyl:CoA ligase
FUMID1_v1_230019	4680494	4681252	automatic/finished	_	Thioesterase superfamily protein
FUMID1_v1_230020	4681302	4682018	automatic/finished	_	Regulatory protein TetR
FUMID1_v1_230024	4685765	4686772	automatic/finished	_	Acyl-CoA dehydrogenase
FUMID1_v1_230025	4686809	4687594	automatic/finished	_	citronellol/citronellal dehydrogenase
FUMID1_v1_230027	4688123	4689106	automatic/finished	_	HTH_30 domain-containing protein
FUMID1_v1_230028	4689035	4689160	automatic/finished	_	protein of unknown function
FUMID1_v1_230029	4689338	4690279	automatic/finished	_	epsilon-lactone hydrolase
FUMID1_v1_230041	4696586	4697872	automatic/finished	_	3-hydroxyisobutyrate dehydrogenase
FUMID1_v1_230042	4697921	4698775	automatic/finished	_	Short-chain dehydrogenase/reductase SDR
FUMID1_v1_230044	4699257	4699937	automatic/finished	_	TetR/AcrR family transcriptional regulator
FUMID1_v1_230045	4700035	4700691	automatic/finished	_	TetR family transcriptional regulator
FUMID1_v1_230046	4700965	4701378	automatic/finished	_	SnoaL-like domain-containing protein
FUMID1_v1_230047	4701375	4702226	automatic/finished	_	NAD(P)-dependent dehydrogenase, short-chain alcohol dehydrogenase family
FUMID1_v1_230060	4713804	4714361	automatic/finished	_	restriction system protein
FUMID1_v1_230061	4714316	4715083	automatic/finished	_	RNA polymerase sigma-70 factor, ECF subfamily
FUMID1_v1_230062	4715080	4715343	automatic/finished	_	putative transmembrane anti-sigma factor
FUMID1_v1_230065	4717839	4718723	automatic/finished	_	conserved membrane protein of unknown function
FUMID1_v1_230067	4719536	4720906	automatic/finished	_	Integral membrane sensor signal transduction histidine kinase
FUMID1_v1_230068	4721124	4723199	automatic/finished	_	Multidrug ABC transporter permease
FUMID1_v1_230070	4723219	4724526	automatic/finished	_	exported protein of unknown function
FUMID1_v1_230080	4731568	4731765	automatic/finished	_	protein of unknown function
FUMID1_v1_230084	4733412	4734833	automatic/finished	_	two-component system, NarL family, sensor histidine kinase DesK
FUMID1_v1_230088	4738230	4738553	automatic/finished	_	conserved protein of unknown function
FUMID1_v1_230094	4746306	4749665	automatic/finished	_	protein of unknown function
FUMID1_v1_230095	4746906	4747157	automatic/finished	_	protein of unknown function
FUMID1_v1_240007	4754104	4756785	automatic/finished	_	Non-specific serine/threonine protein kinase
FUMID1_v1_240022	4772651	4773949	automatic/finished	_	HNHc domain-containing protein
FUMID1_v1_240034	4785327	4785578	automatic/finished	_	conserved protein of unknown function
FUMID1_v1_240035	4785575	4785781	automatic/finished	_	protein of unknown function
FUMID1_v1_240044	4795191	4795316	automatic/finished	_	protein of unknown function
FUMID1_v1_240059	4816424	4816993	automatic/finished	_	protein of unknown function

FUMID1_v1_240062	4820008	4820181	automatic/finished	_	protein of unknown function
FUMID1_v1_250003	4832145	4832759	automatic/finished	_	HTH tetR-type domain-containing protein
FUMID1_v1_250005	4833566	4834075	automatic/finished	_	protein of unknown function
FUMID1_v1_250007	4834036	4835049	automatic/finished	_	L-fuconolactonase
FUMID1_v1_250008	4835162	4836367	automatic/finished	cyp	Cytochrome P450-terp
FUMID1_v1_250016	4843093	4844031	automatic/finished	_	Short-chain dehydrogenase
FUMID1_v1_250017	4844628	4846241	automatic/finished	_	MFS domain-containing protein
FUMID1_v1_250019	4846739	4847971	automatic/finished	_	ABC transporter substrate-binding protein
FUMID1_v1_250020	4847826	4848038	automatic/finished	_	protein of unknown function
FUMID1_v1_250029	4855759	4856031	automatic/finished	_	protein of unknown function
FUMID1_v1_250037	4859703	4861838	automatic/finished	_	Uncharacterized peptidase y4nA
FUMID1_v1_250038	4861801	4862274	automatic/finished	_	exported protein of unknown function
FUMID1_v1_250041	4864995	4866317	automatic/finished	_	GAF domain-containing protein
FUMID1_v1_250046	4869380	4870372	automatic/finished	_	protein of unknown function
FUMID1_v1_250048	4870338	4870631	automatic/finished	_	protein of unknown function
FUMID1_v1_250049	4871731	4872840	automatic/finished	_	Luciferase family protein
FUMID1_v1_250055	4895789	4896871	automatic/finished	_	esterase FrsA
FUMID1_v1_250059	4899952	4900791	automatic/finished	_	peptide/nickel transport system permease protein
FUMID1_v1_250060	4900788	4901762	automatic/finished	_	peptide/nickel transport system permease protein
FUMID1_v1_250061	4901769	4903340	automatic/finished	_	peptide/nickel transport system substrate-binding protein
FUMID1_v1_250062	4903376	4904734	automatic/finished	scmK	N-acetyl-S-(2-succino)cysteine monooxygenase
FUMID1_v1_250065	4907905	4908945	automatic/finished	_	flavin-dependent trigonelline monooxygenase, oxygenase component
FUMID1_v1_250066	4909028	4911022	automatic/finished	_	protein of unknown function
FUMID1_v1_260001	4911362	4911808	automatic/finished	_	exported protein of unknown function
FUMID1_v1_260021	4935181	4935375	automatic/finished	_	protein of unknown function
FUMID1_v1_260023	4935369	4935548	automatic/finished	_	protein of unknown function
FUMID1_v1_260028	4941461	4941775	automatic/finished	_	conserved membrane protein of unknown function
FUMID1_v1_260032	4946921	4947724	automatic/finished	_	exported protein of unknown function
FUMID1_v1_260042	4955043	4955432	automatic/finished	_	protein of unknown function
FUMID1_v1_260068	4984771	4984971	automatic/finished	_	protein of unknown function
FUMID1_v1_270001	4985733	4985933	automatic/finished	_	protein of unknown function
FUMID1_v1_270003	4987278	4987922	automatic/finished	_	N-acetyltransferase
FUMID1_v1_270004	4988002	4988202	automatic/finished	_	protein of unknown function
FUMID1_v1_270007	4989937	4990233	automatic/finished	_	protein of unknown function
FUMID1_v1_270008	4990003	4990224	automatic/finished	_	exported protein of unknown function
FUMID1_v1_270021	4998385	4999578	automatic/finished	_	cytochrome P450 family 130
FUMID1_v1_270022	4999718	5000314	automatic/finished	_	TetR family transcriptional regulator
FUMID1_v1_270025	5003352	5004530	automatic/finished	xylA	Xylose isomerase
FUMID1_v1_270026	5004544	5005986	automatic/finished	xylB	Xylulose kinase
FUMID1_v1_270041	5018685	5020619	automatic/finished	_	protein of unknown function
FUMID1_v1_270042	5020708	5021529	automatic/finished	_	protein of unknown function
FUMID1_v1_270043	5021526	5029220	automatic/finished	_	protein of unknown function
FUMID1_v1_270044	5028113	5029678	automatic/finished	_	protein of unknown function
FUMID1_v1_270046	5029929	5030228	automatic/finished	_	conserved protein of unknown function
FUMID1_v1_270047	5030225	5031409	automatic/finished	_	Transporter (CPA2 family)
FUMID1_v1_270049	5032255	5033067	automatic/finished	_	Enoyl-CoA hydratase/isomerase
FUMID1_v1_270050	5033454	5033582	automatic/finished	_	protein of unknown function
FUMID1_v1_270056	5037857	5038753	automatic/finished	_	xylono-1,5-lactonase
FUMID1_v1_270057	5038750	5040336	automatic/finished	_	Alpha-ketoglutaric semialdehyde dehydrogenase 2
FUMID1_v1_270058	5040381	5041433	automatic/finished	_	Alcohol dehydrogenase GroES domain protein
FUMID1_v1_270059	5041470	5042651	automatic/finished	_	galactonate dehydratase
FUMID1_v1_270060	5042648	5043634	automatic/finished	_	Peptidase family U32
FUMID1_v1_270061	5043922	5045076	automatic/finished	_	LacI family transcriptional regulator
FUMID1_v1_270063	5046449	5047042	automatic/finished	_	AAA family ATPase
FUMID1_v1_270064	5047147	5048595	automatic/finished	_	Exo-alpha-sialidase
FUMID1_v1_270065	5048594	5048701	automatic/finished	_	protein of unknown function
FUMID1_v1_270067	5049481	5050071	automatic/finished	_	TetR family transcriptional regulator
FUMID1_v1_270068	5050148	5051464	automatic/finished	_	Branched-chain amino acid ABC transporter substrate-binding protein
FUMID1_v1_270073	5052241	5053977	automatic/finished	bgxA	Beta-glucosidase / Beta-xylosidase
FUMID1_v1_270074	5054013	5054642	automatic/finished	_	protein of unknown function
FUMID1_v1_280016	5070098	5070760	automatic/finished	_	conserved exported protein of unknown function
FUMID1_v1_280026	5083228	5083686	automatic/finished	_	protein of unknown function
FUMID1_v1_280027	5083724	5084512	automatic/finished	_	TetR family transcriptional regulator
FUMID1_v1_280028	5084670	5085491	automatic/finished	_	Short-chain dehydrogenase/reductase SDR
FUMID1_v1_280029	5085544	5086350	automatic/finished	fabG	3-alpha-(or 20-beta)-hydroxysteroid dehydrogenase
FUMID1_v1_280040	5098567	5098917	automatic/finished	_	protein of unknown function
FUMID1_v1_280051	5107920	5110661	automatic/finished	_	putative Diguanylate cyclase/phosphodiesterase
FUMID1_v1_280055	5113995	5114621	automatic/finished	_	Thioesterase superfamily enzyme
FUMID1_v1_290002	5120923	5121699	automatic/finished	fabG	3-alpha-(or 20-beta)-hydroxysteroid dehydrogenase

FUMID1_v1_290004	5122059	5122397	automatic/finished	_	protein of unknown function
FUMID1_v1_290011	5129645	5130988	automatic/finished	_	HNHc domain-containing protein
FUMID1_v1_290015	5138838	5139947	automatic/finished	_	conserved protein of unknown function
FUMID1_v1_290021	5144197	5145069	automatic/finished	_	exported protein of unknown function
FUMID1_v1_290025	5149535	5150305	automatic/finished	_	SARP family transcriptional regulator, regulator of embCAB operon
FUMID1_v1_290026	5150555	5150872	automatic/finished	_	protein of unknown function
FUMID1_v1_290028	5151600	5151803	automatic/finished	_	protein of unknown function
FUMID1_v1_290029	5151835	5152035	automatic/finished	_	protein of unknown function
FUMID1_v1_290040	5161877	5162272	automatic/finished	_	putative Transcriptional regulator BlaI
FUMID1_v1_290041	5162845	5163966	automatic/finished	_	Peptidase_M48 domain-containing protein
FUMID1_v1_290047	5171142	5172188	automatic/finished	_	iron complex transport system substrate-binding protein
FUMID1_v1_30005	919341	919553	automatic/finished	_	exported protein of unknown function
FUMID1_v1_30030	946953	948926	automatic/finished	_	Calpain catalytic domain-containing protein
FUMID1_v1_30035	955854	957002	automatic/finished	_	protein of unknown function
FUMID1_v1_30038	957872	959011	automatic/finished	_	ElaA protein
FUMID1_v1_30073	990578	991183	automatic/finished	_	TetR family transcriptional regulator
FUMID1_v1_30074	991365	991919	automatic/finished	_	TetR/AcrR family transcriptional regulator
FUMID1_v1_30075	991950	992129	automatic/finished	_	protein of unknown function
FUMID1_v1_30076	992285	994594	automatic/finished	ydfJ	Membrane protein YdfJ
FUMID1_v1_30077	994650	995306	automatic/finished	_	Transcriptional regulator
FUMID1_v1_30081	1001190	1001315	automatic/finished	_	protein of unknown function
FUMID1_v1_30087	1004935	1005363	automatic/finished	_	histidine triad (HIT) family protein
FUMID1_v1_30089	1005900	1006247	automatic/finished	_	protein of unknown function
FUMID1_v1_30090	1006244	1006456	automatic/finished	_	protein of unknown function
FUMID1_v1_30091	1006455	1007075	automatic/finished	_	conserved protein of unknown function
FUMID1_v1_30092	1007182	1007445	automatic/finished	_	protein of unknown function
FUMID1_v1_30093	1007583	1007933	automatic/finished	_	protein of unknown function
FUMID1_v1_30113	1026852	1027157	automatic/finished	_	protein of unknown function
FUMID1_v1_30119	1033822	1034481	automatic/finished	_	protein of unknown function
FUMID1_v1_30130	1049522	1050814	automatic/finished	_	conserved protein of unknown function
FUMID1_v1_30132	1050845	1051018	automatic/finished	_	protein of unknown function
FUMID1_v1_30152	1075373	1076902	automatic/finished	_	exported protein of unknown function
FUMID1_v1_30153	1077096	1077641	automatic/finished	_	protein of unknown function
FUMID1_v1_30154	1077696	1078757	automatic/finished	_	DNA adenine methylase
FUMID1_v1_30155	1078786	1079925	automatic/finished	_	conserved protein of unknown function
FUMID1_v1_30181	1105423	1105698	automatic/finished	_	protein of unknown function
FUMID1_v1_30187	1114940	1115365	automatic/finished	_	conserved exported protein of unknown function
FUMID1_v1_30189	1115523	1115816	automatic/finished	_	protein of unknown function
FUMID1_v1_30195	1122884	1123648	automatic/finished	_	conserved exported protein of unknown function
FUMID1_v1_30217	1145974	1146696	automatic/finished	_	conserved membrane protein of unknown function
FUMID1_v1_30220	1149481	1150158	automatic/finished	_	protein of unknown function
FUMID1_v1_30236	1167308	1167559	automatic/finished	_	protein of unknown function
FUMID1_v1_30239	1170263	1170493	automatic/finished	_	protein of unknown function
FUMID1_v1_30245	1177444	1178346	automatic/finished	_	conserved protein of unknown function
FUMID1_v1_30246	1178365	1178676	automatic/finished	_	protein of unknown function
FUMID1_v1_30263	1191035	1191595	automatic/finished	_	conserved protein of unknown function
FUMID1_v1_30264	1191592	1193607	automatic/finished	_	iron complex transport system ATP-binding protein
FUMID1_v1_30265	1193607	1194734	automatic/finished	_	iron complex transport system permease protein
FUMID1_v1_30266	1194745	1196064	automatic/finished	_	iron complex transport system substrate-binding protein
FUMID1_v1_30270	1197157	1197531	automatic/finished	_	protein of unknown function
FUMID1_v1_30280	1206120	1207706	automatic/finished	fadD	Medium/long-chain-fatty-acid--CoA ligase FadD8
FUMID1_v1_30282	1208690	1209232	automatic/finished	_	conserved protein of unknown function
FUMID1_v1_30283	1209430	1210455	automatic/finished	_	conserved protein of unknown function
FUMID1_v1_30284	1210655	1211986	automatic/finished	_	conserved protein of unknown function
FUMID1_v1_30285	1212127	1213623	automatic/finished	lysS	Lysine--tRNA ligase
FUMID1_v1_30287	1214618	1215163	automatic/finished	_	Calcium-binding protein
FUMID1_v1_30290	1217357	1218136	automatic/finished	_	protein of unknown function
FUMID1_v1_30299	1227460	1229715	automatic/finished	_	protein of unknown function
FUMID1_v1_30301	1229756	1230091	automatic/finished	_	protein of unknown function
FUMID1_v1_30302	1230314	1230460	automatic/finished	_	protein of unknown function
FUMID1_v1_30303	1230272	1230646	automatic/finished	_	protein of unknown function
FUMID1_v1_30304	1230698	1231558	automatic/finished	_	phytanoyl-CoA hydroxylase
FUMID1_v1_30305	1231539	1232522	automatic/finished	_	conserved protein of unknown function
FUMID1_v1_30306	1232539	1233807	automatic/finished	_	Methyltransf_11 domain-containing protein
FUMID1_v1_30307	1233804	1235552	automatic/finished	_	carbamoyltransferase
FUMID1_v1_30308	1235549	1236625	automatic/finished	_	Aldo/keto reductase
FUMID1_v1_30309	1236673	1237911	automatic/finished	_	cysteine-S-conjugate beta-lyase
FUMID1_v1_30310	1237892	1239109	automatic/finished	_	Cytochrome P450
FUMID1_v1_30315	1243821	1244165	automatic/finished	_	protein of unknown function

FUMID1_v1_30317	1244468	1245343	automatic/finished	_	epsilon-lactone hydrolase
FUMID1_v1_30318	1245424	1245663	automatic/finished	_	protein of unknown function
FUMID1_v1_30320	1245847	1246071	automatic/finished	_	protein of unknown function
FUMID1_v1_310001	5243848	5244996	automatic/finished	_	MBL fold metallo-hydrolase
FUMID1_v1_310007	5249561	5251594	automatic/finished	_	conserved protein of unknown function
FUMID1_v1_310008	5251645	5253741	automatic/finished	_	conserved exported protein of unknown function
FUMID1_v1_310010	5254853	5254957	automatic/finished	_	protein of unknown function
FUMID1_v1_310030	5274525	5274998	automatic/finished	_	tRNA_edit domain-containing protein
FUMID1_v1_310031	5274357	5275178	automatic/finished	_	protein of unknown function
FUMID1_v1_310033	5275310	5276827	automatic/finished	_	PPM family protein phosphatase
FUMID1_v1_310036	5279396	5279743	automatic/finished	_	protein of unknown function
FUMID1_v1_310037	5280314	5281723	automatic/finished	_	conserved membrane protein of unknown function
FUMID1_v1_310039	5283930	5284109	automatic/finished	_	protein of unknown function
FUMID1_v1_310052	5299617	5300114	automatic/finished	_	protein of unknown function
FUMID1_v1_310053	5300069	5300836	automatic/finished	_	protein of unknown function
FUMID1_v1_320007	5307199	5308236	automatic/finished	_	conserved exported protein of unknown function
FUMID1_v1_320008	5308450	5308995	automatic/finished	_	conserved exported protein of unknown function
FUMID1_v1_320009	5309310	5309795	automatic/finished	sigL	ECF RNA polymerase sigma factor SigL
FUMID1_v1_320010	5309792	5310442	automatic/finished	_	zf-HC2 domain-containing protein
FUMID1_v1_320012	5311340	5311534	automatic/finished	_	protein of unknown function
FUMID1_v1_320017	5316847	5317836	automatic/finished	_	protein of unknown function
FUMID1_v1_320018	5318000	5318185	automatic/finished	_	protein of unknown function
FUMID1_v1_320047	5350793	5351977	automatic/finished	_	protein of unknown function
FUMID1_v1_320055	5354586	5356001	automatic/finished	_	conserved protein of unknown function
FUMID1_v1_320058	5356309	5356923	automatic/finished	_	conserved membrane protein of unknown function
FUMID1_v1_320059	5356925	5357251	automatic/finished	_	protein of unknown function
FUMID1_v1_320060	5357328	5358749	automatic/finished	_	Glycoside hydrolase family 43 (Modular protein)
FUMID1_v1_330008	5370088	5370441	automatic/finished	_	protein of unknown function
FUMID1_v1_330013	5372459	5374534	automatic/finished	_	protein of unknown function
FUMID1_v1_330015	5376305	5376871	automatic/finished	_	exported protein of unknown function
FUMID1_v1_330051	5416916	5417362	automatic/finished	_	protein of unknown function
FUMID1_v1_340004	5433268	5436102	automatic/finished	_	LuxR family transcriptional regulator, maltose regulon positive regulatory protein
FUMID1_v1_340011	5438587	5439195	automatic/finished	_	conserved protein of unknown function
FUMID1_v1_340012	5439192	5439734	automatic/finished	_	conserved protein of unknown function
FUMID1_v1_340013	5439731	5441131	automatic/finished	_	putative peroxidase
FUMID1_v1_340014	5441171	5441428	automatic/finished	_	protein of unknown function
FUMID1_v1_340015	5441509	5442711	automatic/finished	_	Cytochrome P450
FUMID1_v1_340016	5442938	5443630	automatic/finished	_	TetR family transcriptional regulator
FUMID1_v1_340020	5448106	5448750	automatic/finished	_	protein of unknown function
FUMID1_v1_340038	5463997	5464377	automatic/finished	_	Taurine catabolism dioxygenase TauD, TfdA family
FUMID1_v1_340042	5468453	5472349	automatic/finished	_	Multidrug efflux ATP-binding/permease protein BCG_0231
FUMID1_v1_350025	5495408	5495545	automatic/finished	_	protein of unknown function
FUMID1_v1_350027	5497459	5497644	automatic/finished	_	protein of unknown function
FUMID1_v1_350031	5500468	5500713	automatic/finished	_	protein of unknown function
FUMID1_v1_350033	5501258	5501452	automatic/finished	_	protein of unknown function
FUMID1_v1_350034	5501474	5502382	automatic/finished	_	LLM class F420-dependent oxidoreductase
FUMID1_v1_350038	5506631	5507404	automatic/finished	_	Dehydrogenase
FUMID1_v1_350039	5507429	5507860	automatic/finished	_	protein of unknown function
FUMID1_v1_350040	5507857	5508741	automatic/finished	_	conserved protein of unknown function
FUMID1_v1_350041	5508392	5510944	automatic/finished	_	protein of unknown function
FUMID1_v1_350042	5508751	5509767	automatic/finished	_	conserved protein of unknown function
FUMID1_v1_350043	5511206	5513698	automatic/finished	_	exported protein of unknown function
FUMID1_v1_350045	5514291	5514620	automatic/finished	_	protein of unknown function
FUMID1_v1_350047	5515065	5515592	automatic/finished	_	protein of unknown function
FUMID1_v1_350049	5515605	5515886	automatic/finished	_	protein of unknown function
FUMID1_v1_360002	5526088	5526738	automatic/finished	_	protein of unknown function
FUMID1_v1_360003	5526839	5527969	automatic/finished	_	FG-GAP repeat protein
FUMID1_v1_360005	5530434	5531117	automatic/finished	_	conserved protein of unknown function
FUMID1_v1_360009	5531163	5535200	automatic/finished	_	conserved protein of unknown function
FUMID1_v1_360010	5535197	5537578	automatic/finished	_	conserved protein of unknown function
FUMID1_v1_360011	5537581	5541435	automatic/finished	_	conserved protein of unknown function
FUMID1_v1_360012	5541500	5542960	automatic/finished	_	conserved protein of unknown function
FUMID1_v1_360018	5548275	5549192	automatic/finished	_	Alcohol dehydrogenase GroES domain protein
FUMID1_v1_360026	5554162	5555019	automatic/finished	_	membrane protein of unknown function
FUMID1_v1_360030	5558235	5558537	automatic/finished	_	protein of unknown function
FUMID1_v1_360033	5560912	5562495	automatic/finished	_	peptide/nickel transport system substrate-binding protein
FUMID1_v1_360038	5566674	5567918	automatic/finished	cyp	Pentalenic acid synthase
FUMID1_v1_360039	5567918	5568475	automatic/finished	_	AOC_like domain-containing protein
FUMID1_v1_360040	5568535	5569113	automatic/finished	_	exported protein of unknown function

FUMID1_v1_370023	5588528	5589775	automatic/finished	_	integrase
FUMID1_v1_370024	5590421	5591917	automatic/finished	_	TIR domain-containing protein
FUMID1_v1_370031	5603360	5603752	automatic/finished	_	protein of unknown function
FUMID1_v1_370032	5604126	5605286	automatic/finished	_	conserved protein of unknown function
FUMID1_v1_370033	5603871	5604425	automatic/finished	_	protein of unknown function
FUMID1_v1_370034	5604034	5604102	automatic/finished	_	protein of unknown function
FUMID1_v1_370035	5604923	5605336	automatic/finished	_	protein of unknown function
FUMID1_v1_370038	5606658	5606846	automatic/finished	_	protein of unknown function
FUMID1_v1_370042	5611083	5611331	automatic/finished	_	protein of unknown function
FUMID1_v1_370046	5612919	5613605	automatic/finished	_	protein of unknown function
FUMID1_v1_370047	5613665	5615119	automatic/finished	_	conserved protein of unknown function
FUMID1_v1_380008	5624108	5625733	automatic/finished	_	conserved protein of unknown function
FUMID1_v1_380015	5630848	5631429	automatic/finished	_	protein of unknown function
FUMID1_v1_380017	5632774	5633901	automatic/finished	_	conserved protein of unknown function
FUMID1_v1_380022	5636351	5637094	automatic/finished	_	transcriptional regulator
FUMID1_v1_380029	5643228	5643620	automatic/finished	_	MarR family transcriptional regulator
FUMID1_v1_380032	5645980	5646084	automatic/finished	_	protein of unknown function
FUMID1_v1_380033	5646113	5646643	automatic/finished	_	protein of unknown function
FUMID1_v1_380034	5646276	5646626	automatic/finished	_	protein of unknown function
FUMID1_v1_390035	5700776	5701609	automatic/finished	_	protein of unknown function
FUMID1_v1_400011	5716812	5717171	automatic/finished	_	protein of unknown function
FUMID1_v1_400013	5720152	5721717	automatic/finished	_	membrane protein of unknown function
FUMID1_v1_400014	5721708	5722067	automatic/finished	_	conserved protein of unknown function
FUMID1_v1_400015	5722241	5722549	automatic/finished	_	protein of unknown function
FUMID1_v1_400017	5722876	5724336	automatic/finished	_	conserved membrane protein of unknown function
FUMID1_v1_40008	1264311	1264793	automatic/finished	_	protein of unknown function
FUMID1_v1_40011	1267262	1267438	automatic/finished	_	protein of unknown function
FUMID1_v1_40012	1267265	1267375	automatic/finished	_	protein of unknown function
FUMID1_v1_40066	1326997	1327275	automatic/finished	_	protein of unknown function
FUMID1_v1_40074	1336424	1336558	automatic/finished	_	protein of unknown function
FUMID1_v1_40082	1348011	1348736	automatic/finished	_	protein of unknown function
FUMID1_v1_40099	1364139	1364354	automatic/finished	_	protein of unknown function
FUMID1_v1_40102	1365394	1365570	automatic/finished	_	protein of unknown function
FUMID1_v1_40104	1367558	1367764	automatic/finished	_	protein of unknown function
FUMID1_v1_40108	1370150	1370494	automatic/finished	_	protein of unknown function
FUMID1_v1_40113	1377742	1378281	automatic/finished	_	conserved exported protein of unknown function
FUMID1_v1_40119	1384274	1385680	automatic/finished	_	HNHc domain-containing protein
FUMID1_v1_40129	1396836	1397009	automatic/finished	_	protein of unknown function
FUMID1_v1_40144	1419794	1420111	automatic/finished	_	protein of unknown function
FUMID1_v1_40166	1443292	1444491	automatic/finished	_	Cytochrome P450
FUMID1_v1_40167	1444667	1445512	automatic/finished	_	protein of unknown function
FUMID1_v1_40168	1445552	1446190	automatic/finished	_	HTH tetR-type domain-containing protein
FUMID1_v1_40169	1446196	1446687	automatic/finished	_	SnoaL-like domain-containing protein
FUMID1_v1_40170	1446698	1447093	automatic/finished	_	SnoaL-like domain-containing protein
FUMID1_v1_40171	1447304	1448587	automatic/finished	_	Acyl-CoA dehydrogenase domain protein
FUMID1_v1_40172	1448584	1449735	automatic/finished	_	Acyl-CoA dehydrogenase domain protein
FUMID1_v1_40173	1450176	1451390	automatic/finished	_	Carnitine dehydratase
FUMID1_v1_40175	1453095	1454306	automatic/finished	_	Amidohydrolase 2
FUMID1_v1_40176	1454539	1455657	automatic/finished	_	Acyl-CoA dehydrogenase
FUMID1_v1_40177	1455647	1456855	automatic/finished	_	Acyl-CoA dehydrogenase
FUMID1_v1_40178	1456924	1458018	automatic/finished	_	5,10-methylenetetrahydromethanopterin reductase
FUMID1_v1_40180	1459960	1460868	automatic/finished	_	Amidohydrolase 2
FUMID1_v1_40182	1462053	1463261	automatic/finished	_	Acyl-CoA dehydrogenase
FUMID1_v1_40189	1466090	1466974	automatic/finished	_	Luciferase
FUMID1_v1_40190	1467042	1467167	automatic/finished	_	protein of unknown function
FUMID1_v1_40191	1467238	1468989	automatic/finished	casI	Steroid-22-oyl-CoA synthetase
FUMID1_v1_40193	1468998	1470008	automatic/finished	_	putative coenzyme F420-dependent oxidoreductase Rv3520c
FUMID1_v1_40197	1474485	1475282	automatic/finished	echA	putative enoyl-CoA hydratase EchA13
FUMID1_v1_40198	1475409	1476854	automatic/finished	_	Acyl-CoA synthetase (AMP-forming)/AMP-acid ligase II
FUMID1_v1_40199	1476934	1478112	automatic/finished	_	Amidohydrolase 2
FUMID1_v1_40200	1478164	1479141	automatic/finished	_	Acyl-CoA dehydrogenase
FUMID1_v1_40201	1479138	1480298	automatic/finished	_	Acyl-CoA dehydrogenase domain protein
FUMID1_v1_40203	1481161	1482813	automatic/finished	_	AMP-dependent synthetase and ligase
FUMID1_v1_40204	1482863	1484647	automatic/finished	_	GGDEF domain-containing protein
FUMID1_v1_40205	1484787	1486124	automatic/finished	_	Acyl-CoA thioesterase
FUMID1_v1_40206	1486129	1487283	automatic/finished	_	Acyl-coenzyme A thioesterase PaaI, contains HGG motif
FUMID1_v1_40209	1488780	1489595	automatic/finished	_	3-alpha-(or 20-beta)-hydroxysteroid dehydrogenase
FUMID1_v1_40210	1489687	1490910	automatic/finished	_	Amidohydrolase 2
FUMID1_v1_40211	1490974	1492698	automatic/finished	_	N-acyl-D-amino-acid deacylase

FUMID1_v1_40212	1493013	1494944	automatic/finished	_	4-hydroxyacetophenone monooxygenase
FUMID1_v1_40213	1495012	1495710	automatic/finished	_	Regulatory protein, tetR family
FUMID1_v1_40214	1495703	1497076	automatic/finished	_	Lysophospholipase L1-like esterase
FUMID1_v1_40227	1509473	1509679	automatic/finished	_	protein of unknown function
FUMID1_v1_40251	1528962	1529405	automatic/finished	_	protein of unknown function
FUMID1_v1_40252	1529460	1530392	automatic/finished	_	acetyl esterase
FUMID1_v1_40253	1530650	1530985	automatic/finished	_	protein of unknown function
FUMID1_v1_40257	1532819	1533250	automatic/finished	_	protein of unknown function
FUMID1_v1_40272	1552020	1553975	automatic/finished	_	putative DUF5667 domain-containing protein
FUMID1_v1_40278	1559849	1559968	automatic/finished	_	protein of unknown function
FUMID1_v1_410003	5745882	5746952	automatic/finished	_	NADPH2:quinone reductase
FUMID1_v1_410017	5756702	5757514	automatic/finished	_	protein of unknown function
FUMID1_v1_410019	5757537	5758991	automatic/finished	_	Amino acid permease-associated region
FUMID1_v1_410026	5764416	5764586	automatic/finished	_	protein of unknown function
FUMID1_v1_410027	5764579	5765031	automatic/finished	_	conserved protein of unknown function
FUMID1_v1_410040	5773758	5773973	automatic/finished	_	protein of unknown function
FUMID1_v1_410042	5774884	5775147	automatic/finished	_	protein of unknown function
FUMID1_v1_410045	5776389	5777336	automatic/finished	_	conserved membrane protein of unknown function
FUMID1_v1_410046	5777481	5778770	automatic/finished	_	two-component system, NarL family, sensor histidine kinase DesK
FUMID1_v1_420007	5793320	5793622	automatic/finished	_	protein of unknown function
FUMID1_v1_420008	5793833	5795809	automatic/finished	_	PepX_C domain-containing protein
FUMID1_v1_420009	5793688	5794086	automatic/finished	_	protein of unknown function
FUMID1_v1_420010	5796010	5797206	automatic/finished	serA	phosphoglycerate dehydrogenase
FUMID1_v1_420015	5802264	5803340	automatic/finished	_	5,10-methylenetetrahydromethanopterin reductase
FUMID1_v1_420016	5803370	5804134	automatic/finished	_	TetR family transcriptional regulator
FUMID1_v1_420017	5804261	5805988	automatic/finished	_	N-acyl-D-amino-acid deacylase
FUMID1_v1_420018	5806109	5807335	automatic/finished	_	Amidohydrolase
FUMID1_v1_420019	5807418	5808293	automatic/finished	_	putative F420-dependent oxidoreductase
FUMID1_v1_420020	5808290	5809075	automatic/finished	_	Short-chain dehydrogenase
FUMID1_v1_420021	5809072	5809584	automatic/finished	_	SnoaL-like domain-containing protein
FUMID1_v1_420022	5809712	5810437	automatic/finished	_	conserved protein of unknown function
FUMID1_v1_420023	5810412	5811119	automatic/finished	_	TetR family transcriptional regulator
FUMID1_v1_420024	5811221	5812648	automatic/finished	_	putative NB-ARC domain-containing protein
FUMID1_v1_420037	5824301	5825101	automatic/finished	_	phosphate transport system substrate-binding protein
FUMID1_v1_430003	5829058	5829543	automatic/finished	_	conserved protein of unknown function
FUMID1_v1_430010	5836581	5836850	automatic/finished	_	protein of unknown function
FUMID1_v1_430013	5838071	5838256	automatic/finished	_	protein of unknown function
FUMID1_v1_430025	5851400	5851618	automatic/finished	_	protein of unknown function
FUMID1_v1_430027	5853734	5854498	automatic/finished	_	putative nucleic-acid-binding protein containing a Zn-ribbon
FUMID1_v1_450009	5899115	5902786	automatic/finished	_	Glycosyltransferase like family 2
FUMID1_v1_450018	5912752	5913426	automatic/finished	_	protein of unknown function
FUMID1_v1_460004	5929263	5929427	automatic/finished	_	protein of unknown function
FUMID1_v1_460016	5941803	5942603	automatic/finished	_	Purine nucleoside phosphorylase
FUMID1_v1_460020	5946469	5947377	automatic/finished	_	SDR family NAD(P)-dependent oxidoreductase
FUMID1_v1_460021	5946200	5946418	automatic/finished	_	protein of unknown function
FUMID1_v1_460022	5947597	5948232	automatic/finished	_	protein of unknown function
FUMID1_v1_460025	5948760	5949341	automatic/finished	_	protein of unknown function
FUMID1_v1_460026	5949386	5949565	automatic/finished	_	protein of unknown function
FUMID1_v1_480017	5993133	5994263	automatic/finished	_	2-polyprenyl-6-methoxyphenol hydroxylase
FUMID1_v1_480018	5994294	5995223	automatic/finished	_	putative Biphenyl-2,3-diol 1,2-dioxygenase
FUMID1_v1_480019	5995432	5996406	automatic/finished	_	FAA_hydrolase domain-containing protein
FUMID1_v1_480021	5997728	5998735	automatic/finished	_	APH domain-containing protein
FUMID1_v1_480022	5998732	5999763	automatic/finished	gyaR	Glyoxylate reductase
FUMID1_v1_490003	6021662	6024406	automatic/finished	_	protein of unknown function
FUMID1_v1_500001	6024574	6032586	automatic/finished	_	protein of unknown function
FUMID1_v1_500009	6042826	6044061	automatic/finished	_	Amidohydrolase 2
FUMID1_v1_500010	6044335	6045045	automatic/finished	_	TetR/AcrR family transcriptional regulator, tetracycline repressor protein
FUMID1_v1_50013	1575549	1575953	automatic/finished	_	protein of unknown function
FUMID1_v1_50020	1579601	1580665	automatic/finished	_	2-polyprenyl-6-methoxyphenol hydroxylase-like FAD-dependent oxidoreductase
FUMID1_v1_50021	1580708	1581268	automatic/finished	_	protein of unknown function
FUMID1_v1_50022	1581307	1582164	automatic/finished	_	exodeoxyribonuclease III
FUMID1_v1_50023	1581316	1582605	automatic/finished	_	protein of unknown function
FUMID1_v1_50030	1586799	1587290	automatic/finished	_	protein of unknown function
FUMID1_v1_50031	1587872	1588783	automatic/finished	_	NADPH:quinone reductase
FUMID1_v1_50032	1588876	1589082	automatic/finished	_	protein of unknown function
FUMID1_v1_50033	1589210	1590925	automatic/finished	_	conserved protein of unknown function
FUMID1_v1_50055	1606696	1607538	automatic/finished	_	XRE family transcriptional regulator
FUMID1_v1_50056	1607576	1607653	automatic/finished	_	protein of unknown function
FUMID1_v1_50060	1610576	1610932	automatic/finished	_	conserved protein of unknown function

FUMID1_v1_50062	1611493	1612245	automatic/finished	_	protein of unknown function
FUMID1_v1_50066	1614437	1615315	automatic/finished	_	DNA-binding protein
FUMID1_v1_50067	1615430	1617070	automatic/finished	mdtD	putative multidrug resistance protein MdtD
FUMID1_v1_50068	1617193	1617408	automatic/finished	_	protein of unknown function
FUMID1_v1_50069	1617396	1618265	automatic/finished	_	Alpha/beta hydrolase
FUMID1_v1_50070	1618265	1618813	automatic/finished	_	Transcriptional regulator, TetR family
FUMID1_v1_50071	1619016	1620245	automatic/finished	_	putative RNA polymerase, sigma 70 family subunit
FUMID1_v1_50073	1620242	1620652	automatic/finished	_	DGPFAETKE family protein
FUMID1_v1_50081	1630208	1631752	automatic/finished	_	tyrosinase
FUMID1_v1_50082	1632005	1632211	automatic/finished	_	protein of unknown function
FUMID1_v1_50084	1637889	1638467	automatic/finished	_	protein of unknown function
FUMID1_v1_50086	1638723	1639127	automatic/finished	_	conserved exported protein of unknown function
FUMID1_v1_50087	1639210	1640250	automatic/finished	_	conserved exported protein of unknown function
FUMID1_v1_50088	1640481	1640939	automatic/finished	_	Transcriptional regulator, MarR family
FUMID1_v1_50094	1645552	1645788	automatic/finished	_	protein of unknown function
FUMID1_v1_50100	1655192	1656031	automatic/finished	_	putative Metal-binding integral membrane protein-like protein
FUMID1_v1_50101	1656135	1657691	automatic/finished	_	Tyrosinase
FUMID1_v1_50103	1658114	1658971	automatic/finished	_	NADP-dependent 3-hydroxy acid dehydrogenase YdfG
FUMID1_v1_50104	1659151	1659861	automatic/finished	_	Short-chain dehydrogenase
FUMID1_v1_50106	1660809	1661360	automatic/finished	_	protein of unknown function
FUMID1_v1_50107	1661458	1661931	automatic/finished	_	protein of unknown function
FUMID1_v1_50113	1669540	1669884	automatic/finished	_	protein of unknown function
FUMID1_v1_50140	1696924	1697115	automatic/finished	_	protein of unknown function
FUMID1_v1_50141	1697172	1698089	automatic/finished	_	conserved protein of unknown function
FUMID1_v1_50144	1699687	1699971	automatic/finished	_	protein of unknown function
FUMID1_v1_50151	1706862	1706930	automatic/finished	_	protein of unknown function
FUMID1_v1_50156	1734484	1734681	automatic/finished	_	protein of unknown function
FUMID1_v1_50162	1744838	1745023	automatic/finished	_	protein of unknown function
FUMID1_v1_50170	1752282	1752935	automatic/finished	_	protein of unknown function
FUMID1_v1_50171	1753124	1753363	automatic/finished	_	protein of unknown function
FUMID1_v1_50179	1759928	1760194	automatic/finished	_	protein of unknown function
FUMID1_v1_50182	1760761	1760907	automatic/finished	_	protein of unknown function
FUMID1_v1_50184	1762026	1762544	automatic/finished	_	exported protein of unknown function
FUMID1_v1_50205	1779840	1780352	automatic/finished	_	conserved protein of unknown function
FUMID1_v1_50206	1780384	1781055	automatic/finished	_	conserved exported protein of unknown function
FUMID1_v1_50223	1807809	1808462	automatic/finished	_	conserved protein of unknown function
FUMID1_v1_50232	1815756	1816049	automatic/finished	_	protein of unknown function
FUMID1_v1_50235	1818155	1818367	automatic/finished	_	protein of unknown function
FUMID1_v1_50236	1818265	1818483	automatic/finished	_	protein of unknown function
FUMID1_v1_50237	1818644	1819165	automatic/finished	_	protein of unknown function
FUMID1_v1_50243	1823358	1823759	automatic/finished	_	protein of unknown function
FUMID1_v1_50249	1829005	1829256	automatic/finished	_	protein of unknown function
FUMID1_v1_50251	1830461	1831855	automatic/finished	_	Chromosome segregation protein SMC
FUMID1_v1_50254	1833826	1834041	automatic/finished	_	protein of unknown function
FUMID1_v1_50270	1850442	1850534	automatic/finished	_	protein of unknown function
FUMID1_v1_510005	6052660	6052773	automatic/finished	_	protein of unknown function
FUMID1_v1_510006	6052852	6053853	automatic/finished	_	Luciferase
FUMID1_v1_510007	6053760	6054146	automatic/finished	_	protein of unknown function
FUMID1_v1_510011	6056480	6056728	automatic/finished	_	protein of unknown function
FUMID1_v1_510012	6056709	6057839	automatic/finished	_	2-polyprenyl-6-methoxyphenol hydroxylase
FUMID1_v1_510014	6058469	6058780	automatic/finished	_	protein of unknown function
FUMID1_v1_520004	6074340	6076313	automatic/finished	_	conserved exported protein of unknown function
FUMID1_v1_520011	6080607	6081404	automatic/finished	_	conserved protein of unknown function
FUMID1_v1_520012	6081401	6082375	automatic/finished	_	Rubrerythrin family protein
FUMID1_v1_520016	6086049	6086540	automatic/finished	_	VOC domain-containing protein
FUMID1_v1_520017	6086537	6086899	automatic/finished	_	Glyoxalase
FUMID1_v1_520018	6087123	6087866	automatic/finished	_	putative FHA domain-containing protein
FUMID1_v1_520020	6089305	6089796	automatic/finished	_	protein of unknown function
FUMID1_v1_520023	6093181	6095349	automatic/finished	_	beta-glucosidase
FUMID1_v1_530002	6097052	6097888	automatic/finished	_	protein of unknown function
FUMID1_v1_530003	6098655	6099866	automatic/finished	cyp	Steroid C26-monooxygenase
FUMID1_v1_530004	6099942	6100739	automatic/finished	_	enoyl-CoA hydratase
FUMID1_v1_530005	6100834	6101973	automatic/finished	_	Phenylpropionate dioxygenase, large terminal subunit
FUMID1_v1_530006	6102139	6103038	automatic/finished	_	putative short-chain type dehydrogenase/reductase Rv0148
FUMID1_v1_530007	6103142	6104701	automatic/finished	fadD	Medium/long-chain-fatty-acid--CoA ligase FadD8
FUMID1_v1_530009	6106313	6107431	automatic/finished	adhB	Alcohol dehydrogenase B
FUMID1_v1_530010	6107474	6108463	automatic/finished	_	OB_aCoA_assoc domain-containing protein
FUMID1_v1_530011	6108463	6109515	automatic/finished	_	Lipid-transfer protein
FUMID1_v1_530012	6109512	6110672	automatic/finished	_	Thiolase domain-containing protein

FUMID1_v1_530015	6111521	6113176	automatic/finished	fadD	Medium/long-chain-fatty-acid--CoA/3-oxocholest-4-en-26-oate--CoA ligase
FUMID1_v1_530017	6113915	6114385	automatic/finished	_	Glyoxalase/bleomycin resistance protein/dioxygenase
FUMID1_v1_530018	6114641	6114997	automatic/finished	_	exported protein of unknown function
FUMID1_v1_530019	6115109	6115834	automatic/finished	_	putative hydrolase of the HAD superfamily
FUMID1_v1_530020	6116455	6116955	automatic/finished	_	Hemerythrin
FUMID1_v1_540009	6122442	6123536	automatic/finished	_	conserved protein of unknown function
FUMID1_v1_540010	6123538	6124500	automatic/finished	_	Malonyl transferase
FUMID1_v1_540012	6125382	6128192	automatic/finished	_	protein of unknown function
FUMID1_v1_560002	6160392	6160778	automatic/finished	glxB	methylglyoxalase; lactoylbacillithiol lyase
FUMID1_v1_560003	6160885	6161097	automatic/finished	_	protein of unknown function
FUMID1_v1_560004	6161253	6162083	automatic/finished	_	putative non-heme chloroperoxidase
FUMID1_v1_560009	6172112	6172480	automatic/finished	_	protein of unknown function
FUMID1_v1_560012	6174775	6176241	automatic/finished	_	conserved protein of unknown function
FUMID1_v1_560013	6176238	6177257	automatic/finished	_	membrane protein of unknown function
FUMID1_v1_570001	6177529	6178770	automatic/finished	_	Peripla_BP_6 domain-containing protein
FUMID1_v1_570002	6178969	6181383	automatic/finished	_	beta-glucosidase
FUMID1_v1_570003	6181412	6181654	automatic/finished	_	protein of unknown function
FUMID1_v1_570004	6181674	6181892	automatic/finished	_	protein of unknown function
FUMID1_v1_570005	6182061	6182945	automatic/finished	nlhH	Carboxylesterase NlhH
FUMID1_v1_570006	6182971	6184122	automatic/finished	galK	Galactokinase
FUMID1_v1_570007	6184119	6185093	automatic/finished	_	Galactose-1-phosphate uridylyltransferase
FUMID1_v1_570008	6185090	6185314	automatic/finished	_	protein of unknown function
FUMID1_v1_570009	6185781	6188282	automatic/finished	_	beta-galactosidase
FUMID1_v1_570011	6188446	6189663	automatic/finished	_	putative Peripla_BP_6 domain-containing protein
FUMID1_v1_570014	6193286	6194008	automatic/finished	_	TetR/AcrR family transcriptional regulator, tetracycline repressor protein
FUMID1_v1_570015	6194005	6194643	automatic/finished	_	platelet-activating factor acetylhydrolase IB subunit beta/gamma
FUMID1_v1_580014	6211160	6211252	automatic/finished	_	protein of unknown function
FUMID1_v1_590001	6211420	6211557	automatic/finished	_	protein of unknown function
FUMID1_v1_590004	6214218	6215771	automatic/finished	_	Aldehyde dehydrogenase (NAD(+))
FUMID1_v1_590007	6217745	6217969	automatic/finished	_	protein of unknown function
FUMID1_v1_590008	6218028	6218168	automatic/finished	_	protein of unknown function
FUMID1_v1_590009	6218554	6218952	automatic/finished	_	protein of unknown function
FUMID1_v1_590010	6219002	6219799	automatic/finished	_	NADPH-dependent ferric siderophore reductase
FUMID1_v1_590011	6219924	6220196	automatic/finished	_	protein of unknown function
FUMID1_v1_590012	6219872	6220213	automatic/finished	_	protein of unknown function
FUMID1_v1_590015	6221691	6223184	automatic/finished	_	2-polyprenyl-6-methoxyphenol hydroxylase-like FAD-dependent oxidoreductase
FUMID1_v1_590016	6223392	6224357	automatic/finished	_	DNA-binding Lrp family transcriptional regulator
FUMID1_v1_600004	6231502	6233241	automatic/finished	_	Dyp-type peroxidase family
FUMID1_v1_600005	6233238	6234404	automatic/finished	_	conserved protein of unknown function
FUMID1_v1_60003	1864718	1865011	automatic/finished	_	protein of unknown function
FUMID1_v1_60014	1876593	1877876	automatic/finished	_	Glycosyltransferase involved in cell wall bisynthesis
FUMID1_v1_60015	1877909	1879285	automatic/finished	_	conserved protein of unknown function
FUMID1_v1_60016	1879318	1880364	automatic/finished	_	vancomycin resistance protein VanJ
FUMID1_v1_60017	1880370	1882355	automatic/finished	_	membrane protein of unknown function
FUMID1_v1_60018	1882611	1882907	automatic/finished	_	protein of unknown function
FUMID1_v1_60020	1883337	1883420	automatic/finished	_	protein of unknown function
FUMID1_v1_60021	1883643	1884536	automatic/finished	_	dolichol-phosphate hexosyltransferase
FUMID1_v1_60022	1884649	1887261	automatic/finished	_	conserved membrane protein of unknown function
FUMID1_v1_60023	1887258	1888466	automatic/finished	_	Glycosyltransferase involved in cell wall biosynthesis
FUMID1_v1_60024	1888463	1889179	automatic/finished	_	Polysaccharide deacetylase
FUMID1_v1_60025	1889176	1889697	automatic/finished	_	protein of unknown function
FUMID1_v1_60026	1889792	1891039	automatic/finished	_	protein of unknown function
FUMID1_v1_60027	1891000	1892364	automatic/finished	_	polysaccharide biosynthesis protein PslG
FUMID1_v1_60028	1892484	1893869	automatic/finished	_	Dihydrolipoamide acetyltransferase component of pyruvate dehydrogenase complex
FUMID1_v1_60031	1896209	1897387	automatic/finished	_	prephenate dehydrogenase
FUMID1_v1_60032	1897731	1898432	automatic/finished	_	maltose O-acetyltransferase
FUMID1_v1_60033	1898429	1899508	automatic/finished	_	glucosyl-dolichyl phosphate glucuronosyltransferase
FUMID1_v1_60034	1899579	1901135	automatic/finished	_	Polysaccharide biosynthesis protein
FUMID1_v1_60036	1902319	1902984	automatic/finished	_	membrane protein of unknown function
FUMID1_v1_60037	1903046	1905253	automatic/finished	_	conserved membrane protein of unknown function
FUMID1_v1_60040	1907776	1908045	automatic/finished	_	protein of unknown function
FUMID1_v1_60041	1908042	1908668	automatic/finished	_	maltose O-acetyltransferase
FUMID1_v1_60043	1909312	1910898	automatic/finished	_	Carboxylic ester hydrolase
FUMID1_v1_60046	1912768	1914210	automatic/finished	_	4-hydroxyphenylacetate 3-monooxygenase
FUMID1_v1_60048	1915216	1916727	automatic/finished	_	Histidine kinase
FUMID1_v1_60109	1984300	1984707	automatic/finished	_	protein of unknown function
FUMID1_v1_60127	2004155	2004394	automatic/finished	_	protein of unknown function
FUMID1_v1_60169	2053623	2053997	automatic/finished	_	exported protein of unknown function
FUMID1_v1_60170	2053930	2054139	automatic/finished	_	protein of unknown function

FUMID1_v1_60191	2074186	2075049	automatic/finished	_	3-oxoacyl-ACP reductase
FUMID1_v1_60194	2076628	2076951	automatic/finished	_	Antibiotic biosynthesis monooxygenase
FUMID1_v1_60200	2079481	2080818	automatic/finished	_	Branched-chain amino acid ABC transporter substrate-binding protein
FUMID1_v1_60201	2080043	2080873	automatic/finished	_	exported protein of unknown function
FUMID1_v1_60205	2086994	2089543	automatic/finished	_	protein-glutamine gamma-glutamyltransferase
FUMID1_v1_60248	2128433	2128615	automatic/finished	_	protein of unknown function
FUMID1_v1_610001	6243595	6245268	automatic/finished	_	AMP-dependent synthetase and ligase
FUMID1_v1_610004	6247098	6247715	automatic/finished	_	DinB family protein
FUMID1_v1_610005	6247777	6248781	automatic/finished	_	Transcriptional regulator
FUMID1_v1_610006	6248778	6251408	automatic/finished	_	protein of unknown function
FUMID1_v1_610007	6251410	6252732	automatic/finished	_	Low temperature requirement A
FUMID1_v1_620001	6259107	6259463	automatic/finished	_	conserved protein of unknown function
FUMID1_v1_620007	6268907	6269380	automatic/finished	_	protein of unknown function
FUMID1_v1_620012	6271787	6272806	automatic/finished	_	protein of unknown function
FUMID1_v1_640001	6283638	6284549	automatic/finished	_	membrane protein of unknown function
FUMID1_v1_640002	6284546	6286174	automatic/finished	_	protein of unknown function
FUMID1_v1_640003	6286224	6287075	automatic/finished	_	protein of unknown function
FUMID1_v1_640007	6291827	6292864	automatic/finished	_	protein of unknown function
FUMID1_v1_660001	6302150	6302614	automatic/finished	_	conserved protein of unknown function
FUMID1_v1_660002	6302682	6302807	automatic/finished	_	protein of unknown function
FUMID1_v1_660003	6302902	6303084	automatic/finished	_	protein of unknown function
FUMID1_v1_660004	6302956	6303051	automatic/finished	_	protein of unknown function
FUMID1_v1_660005	6303077	6303202	automatic/finished	_	protein of unknown function
FUMID1_v1_660007	6308063	6309733	automatic/finished	_	choline dehydrogenase
FUMID1_v1_670001	6310714	6311172	automatic/finished	_	protein of unknown function
FUMID1_v1_670002	6310865	6311632	automatic/finished	_	protein of unknown function
FUMID1_v1_670003	6311648	6312520	automatic/finished	_	conserved protein of unknown function
FUMID1_v1_670004	6312853	6313551	automatic/finished	_	fluoroquinolone resistance protein
FUMID1_v1_670009	6315858	6316190	automatic/finished	_	protein of unknown function
FUMID1_v1_670010	6315797	6316447	automatic/finished	_	protein of unknown function
FUMID1_v1_700004	6332418	6333158	automatic/finished	_	protein of unknown function
FUMID1_v1_700005	6332697	6332870	automatic/finished	_	protein of unknown function
FUMID1_v1_70001	2152053	2152301	automatic/finished	_	protein of unknown function
FUMID1_v1_70004	2153968	2154192	automatic/finished	_	protein of unknown function
FUMID1_v1_70005	2154706	2154981	automatic/finished	_	protein of unknown function
FUMID1_v1_70007	2155069	2156304	automatic/finished	_	ABC transporter substrate-binding protein
FUMID1_v1_70008	2156711	2157940	automatic/finished	_	Peripla_BP_6 domain-containing protein
FUMID1_v1_70009	2158092	2158637	automatic/finished	_	SnoaL-like domain-containing protein
FUMID1_v1_70011	2159901	2161130	automatic/finished	_	Peripla_BP_6 domain-containing protein
FUMID1_v1_70012	2159840	2160016	automatic/finished	_	protein of unknown function
FUMID1_v1_70017	2166775	2167392	automatic/finished	_	Flavodoxin domain
FUMID1_v1_70036	2185850	2186374	automatic/finished	_	protein of unknown function
FUMID1_v1_70046	2196378	2196713	automatic/finished	_	protein of unknown function
FUMID1_v1_70066	2210611	2211948	automatic/finished	_	threonyl-tRNA synthetase
FUMID1_v1_70071	2220571	2222373	automatic/finished	_	TIR domain-containing protein
FUMID1_v1_70072	2222189	2222638	automatic/finished	_	protein of unknown function
FUMID1_v1_70086	2242834	2243310	automatic/finished	_	protein of unknown function
FUMID1_v1_70092	2246559	2247020	automatic/finished	_	protein of unknown function
FUMID1_v1_70096	2250744	2252174	automatic/finished	_	secreted effector protein PipB2
FUMID1_v1_70098	2252899	2254455	automatic/finished	_	MFS transporter, DHA2 family, multidrug resistance protein
FUMID1_v1_70099	2254460	2254975	automatic/finished	_	protein of unknown function
FUMID1_v1_70103	2258353	2258598	automatic/finished	_	protein of unknown function
FUMID1_v1_70104	2258390	2258461	automatic/finished	_	protein of unknown function
FUMID1_v1_70109	2262748	2263887	automatic/finished	_	conserved protein of unknown function
FUMID1_v1_70110	2263661	2264119	automatic/finished	_	protein of unknown function
FUMID1_v1_70117	2269251	2269691	automatic/finished	_	protein of unknown function
FUMID1_v1_70127	2279721	2279900	automatic/finished	_	protein of unknown function
FUMID1_v1_70141	2291066	2291284	automatic/finished	_	protein of unknown function
FUMID1_v1_70149	2298303	2298947	automatic/finished	_	protein of unknown function
FUMID1_v1_70157	2308116	2308424	automatic/finished	_	protein of unknown function
FUMID1_v1_70161	2310365	2311174	automatic/finished	_	protein of unknown function
FUMID1_v1_70163	2312390	2313409	automatic/finished	_	conserved protein of unknown function
FUMID1_v1_70164	2313415	2314305	automatic/finished	_	DUF4097 domain-containing protein
FUMID1_v1_70165	2314394	2315128	automatic/finished	_	Toxin-antitoxin system HicB family antitoxin
FUMID1_v1_70174	2323715	2323984	automatic/finished	_	protein of unknown function
FUMID1_v1_70180	2329502	2329771	automatic/finished	_	protein of unknown function
FUMID1_v1_70182	2329506	2329904	automatic/finished	_	protein of unknown function
FUMID1_v1_70209	2363953	2364114	automatic/finished	_	protein of unknown function
FUMID1_v1_70240	2407527	2407736	automatic/finished	_	protein of unknown function

FUMID1_v1_710005	6338721	6338843	automatic/finished	_	protein of unknown function
FUMID1_v1_770001	6367201	6370389	automatic/finished	_	protein of unknown function
FUMID1_v1_770002	6370424	6371287	automatic/finished	_	protein of unknown function
FUMID1_v1_790006	6378553	6378852	automatic/finished	_	protein of unknown function
FUMID1_v1_80023	2451521	2451775	automatic/finished	_	protein of unknown function
FUMID1_v1_80024	2451910	2452083	automatic/finished	_	protein of unknown function
FUMID1_v1_80062	2490325	2490507	automatic/finished	_	protein of unknown function
FUMID1_v1_80090	2521001	2521129	automatic/finished	_	protein of unknown function
FUMID1_v1_80174	2623298	2623489	automatic/finished	_	protein of unknown function
FUMID1_v1_80181	2629579	2629854	automatic/finished	_	protein of unknown function
FUMID1_v1_80185	2634455	2634718	automatic/finished	_	protein of unknown function
FUMID1_v1_80201	2653411	2654652	automatic/finished	_	conserved exported protein of unknown function
FUMID1_v1_80207	2661225	2661485	automatic/finished	_	protein of unknown function
FUMID1_v1_80210	2663575	2664375	automatic/finished	_	exported protein of unknown function
FUMID1_v1_870001	6395188	6395574	automatic/finished	_	exported protein of unknown function
FUMID1_v1_870003	6396402	6396638	automatic/finished	_	protein of unknown function
FUMID1_v1_90012	2689534	2689920	automatic/finished	_	protein of unknown function
FUMID1_v1_90015	2694120	2694290	automatic/finished	_	protein of unknown function
FUMID1_v1_90016	2694318	2694557	automatic/finished	_	protein of unknown function
FUMID1_v1_90024	2704260	2704958	automatic/finished	_	putative Vitamin B12 ABC transporter, substrate-binding protein BtuF
FUMID1_v1_90028	2707194	2707583	automatic/finished	_	conserved protein of unknown function
FUMID1_v1_90031	2708865	2709152	automatic/finished	_	protein of unknown function
FUMID1_v1_90069	2758624	2758764	automatic/finished	_	protein of unknown function
FUMID1_v1_90074	2763820	2764455	automatic/finished	_	protein of unknown function
FUMID1_v1_90082	2773256	2773978	automatic/finished	_	exported protein of unknown function
FUMID1_v1_90099	2795301	2795627	automatic/finished	_	protein of unknown function
FUMID1_v1_90102	2796862	2798985	automatic/finished	_	Uncharacterized MFS-type transporter Rv1877
FUMID1_v1_90103	2799197	2799637	automatic/finished	_	conserved protein of unknown function
FUMID1_v1_90104	2799770	2800084	automatic/finished	_	conserved protein of unknown function
FUMID1_v1_90112	2809376	2809561	automatic/finished	_	protein of unknown function
FUMID1_v1_90121	2816946	2818010	automatic/finished	_	conserved membrane protein of unknown function
FUMID1_v1_90139	2834095	2835195	automatic/finished	_	protein of unknown function
FUMID1_v1_90157	2852947	2853090	automatic/finished	_	protein of unknown function
FUMID1_v1_90161	2857702	2857902	automatic/finished	_	protein of unknown function
FUMID1_v1_90168	2863128	2863304	automatic/finished	_	protein of unknown function
FUMID1_v1_90178	2875207	2875383	automatic/finished	_	protein of unknown function
FUMID1_v1_930001	6405056	6405172	automatic/finished	_	protein of unknown function
FUMID1_v1_930002	6405227	6406402	automatic/finished	_	transposase
FUMID1_v1_980001	6411831	6412664	automatic/finished	_	protein of unknown function
FUMID1_v1_980002	6412609	6412944	automatic/finished	_	protein of unknown function

Genes of Frankia alni ACN14a with homologs in Frankia sp. QA3, Frankia torreyi CpI1, Frankia canadensis ARgP5 without homologs in Ag45Mut15 and AgPM24
Homology constraints: minLrap ≥ 0.8 ; maxLrap ≥ 0 ; Identity ≥ 50% similarities involved in a Synteny group

459 results

Label	Begin	End	Evidence	Gene	Product
FRAAL0088	88596	89819	validated/inProgress	_	hypothetical protein
FRAAL0090	91201	91689	validated/inProgress	_	hypothetical protein
FRAAL0093	94218	95675	validated/inProgress	_	putative RNA polymerase sigma factor
FRAAL0100	102452	103693	validated/finished	_	Conserved TM helix repeat-containing protein
FRAAL0119	119716	120345	validated/inProgress	_	hypothetical protein
FRAAL0127	127930	128742	validated/finished	_	conserved protein of unknown function
FRAAL0158	153451	154317	validated/inProgress	_	hypothetical protein
FRAAL0170	164784	166976	validated/inProgress	_	putative integral membrane protein
FRAAL0173	171732	172211	validated/inProgress	_	hypothetical protein
FRAAL0175	173059	173580	validated/inProgress	_	putative transcriptional regulator
FRAAL0176	173759	175267	validated/inProgress	_	putative membrane transport protein
FRAAL0177	175624	177327	validated/inProgress	_	Putative transcriptional regulator (GntR-family)
FRAAL0199	195726	196838	validated/inProgress	_	Alcohol dehydrogenase
FRAAL0200	196889	198448	validated/finished	_	NAD+-dependent betaine aldehyde dehydrogenase
FRAAL0268	286455	287486	validated/inProgress	_	conserved hypothetical protein; putative cobalamin synthesis protein.
FRAAL0277	295497	296351	validated/inProgress	surE	acid phosphatase SurE, survival protein.
FRAAL0290	309655	310023	validated/inProgress	_	hypothetical protein
FRAAL0293	312840	314888	validated/inProgress	thrS	Threonyl-tRNA synthetase (Threonine--tRNA ligase)
FRAAL0317	338179	339879	validated/inProgress	_	conserved hypothetical protein; putative endonuclease domain.
FRAAL0321	344209	344997	validated/inProgress	_	short-chain dehydrogenase, SDR family.
FRAAL0346	374291	381133	validated/inProgress	_	putative Type I modular polyketide synthase

FRAAL0347	381168	390587	validated/inProgress	_	putative Type I modular polyketide synthase
FRAAL0357	422435	423043	validated/finished	_	Transcriptional regulator, TetR-family
FRAAL0378	445636	446754	validated/inProgress	_	putative acyl-CoA dehydrogenase
FRAAL0387	457062	457508	validated/finished	_	conserved hypothetical protein
FRAAL0393	462399	462824	validated/inProgress	_	MutT/nudix family protein
FRAAL0400	470336	471010	validated/finished	_	NIPSNAP domain containing protein
FRAAL0401	471032	471658	validated/finished	_	Putative oxidoreductase
FRAAL0402	471755	472417	validated/inProgress	_	putative regulatory protein
FRAAL0406	475771	475986	validated/inProgress	_	conserved hypothetical protein
FRAAL0407	476018	476431	validated/inProgress	_	hypothetical protein
FRAAL0452	519064	521865	validated/finished	_	CRISPR-associated endonuclease/helicase Cas3
FRAAL0482	547196	548044	validated/finished	_	Lysophospholipase
FRAAL0483	548111	549397	validated/finished	_	Cytochrome P450
FRAAL0485	550001	550360	validated/inProgress	_	conserved hypothetical protein
FRAAL0486	550357	550776	validated/inProgress	_	conserved hypothetical protein
FRAAL0487	550805	553048	validated/inProgress	_	putative sensor-like histidine kinase
FRAAL0520	582116	582394	validated/inProgress	_	hypothetical protein
FRAAL0521	582382	583542	validated/inProgress	_	Putative membrane protein (partial); putative signaling pathway G-protein coupled receptor protein
FRAAL0522	583558	583713	validated/inProgress	_	hypothetical protein
FRAAL0533	592585	592902	validated/inProgress	_	conserved hypothetical protein
FRAAL0582	638654	640453	validated/inProgress	_	Putative efflux membrane protein (partial match)
FRAAL0589	644899	645867	validated/inProgress	_	putative alkanesulfonate transport protein (ABC superfamily)
FRAAL0592	647507	648631	validated/inProgress	_	putative ROK-family transcriptional regulator
FRAAL0607	667023	668045	validated/inProgress	_	putative alcohol dehydrogenase
FRAAL0623	684144	684575	validated/inProgress	_	Putative transcriptional regulator
FRAAL0662	722763	723911	validated/finished	_	Heat shock protein DnaJ-like
FRAAL0673	733761	734936	validated/inProgress	_	Putative DeoR family transcriptional regulator
FRAAL0674	735035	735511	validated/inProgress	_	hypothetical protein
FRAAL0708	770017	770952	validated/inProgress	_	putative non-haem bromoperoxidase (Bromide peroxidase)
FRAAL0710	771743	773248	validated/inProgress	_	hypothetical protein; putative signal peptide
FRAAL0716	777869	778285	validated/inProgress	_	hypothetical protein
FRAAL0735	793927	795675	validated/inProgress	_	putative acyl-CoA synthetase (long-chain-fatty-acid--CoA ligase)
FRAAL0755	815411	817264	validated/finished	_	putative protein-tyrosine kinase
FRAAL0756	817342	818328	validated/finished	_	C4-dicarboxylate ABC transporter substrate-binding protein
FRAAL0764	826376	827392	validated/inProgress	_	putative oxidoreductase, NAD(P)-binding domain (partial match)
FRAAL0765	827823	829124	validated/inProgress	_	putative RNA-binding protein
FRAAL0769	834276	834728	validated/inProgress	_	Putative two component system response regulator
FRAAL0770	834912	835985	validated/inProgress	_	putative Ribokinase
FRAAL0771	836225	840175	validated/inProgress	_	putative signaling protein with a PAS domain
FRAAL0780	847610	848044	validated/finished	_	putative ribokinase (C-terminal part)
FRAAL0882	947399	949285	validated/inProgress	_	putative Beta-N-acetylglucosaminidase precursor
FRAAL0890	955919	960916	validated/inProgress	_	hypothetical protein; putative membrane protein
FRAAL0898	968449	969558	validated/finished	_	Diguanylate cyclase with PAS/PAC sensor
FRAAL0899	969948	970643	validated/finished	_	conserved secreted protein of unknown function
FRAAL0923	991978	992346	validated/inProgress	_	hypothetical protein
FRAAL0924	992392	993444	validated/inProgress	_	Putative MarR-family transcriptional regulator; putative signal peptide
FRAAL0932	1000703	1001158	validated/inProgress	_	hypothetical protein
FRAAL0947	1010461	1010562	validated/finished	_	conserved protein of unknown function
FRAAL0958	1020600	1021478	validated/inProgress	_	putative sugar transport system permease protein
FRAAL0959	1021475	1022446	validated/inProgress	_	putative permease protein of sugar ABC transporter precursor
FRAAL0961	1023814	1025088	validated/inProgress	_	putative sugar transport ATP-hydrolyzing subunit (partial match)
FRAAL0995	1062102	1063748	validated/inProgress	_	hypothetical protein; putative membrane protein
FRAAL1009	1080832	1081092	validated/inProgress	_	hypothetical protein
FRAAL1029	1101440	1102156	validated/inProgress	_	Hypothetical protein
FRAAL1143	1207861	1211076	validated/finished	_	conserved membrane protein of unknown function
FRAAL1153	1224371	1225267	validated/finished	_	Putative secreted lipase
FRAAL1172	1244606	1245859	validated/finished	_	Ribonuclease
FRAAL1185	1256920	1259220	validated/inProgress	_	putative ATP-dependent DNA helicase
FRAAL1186	1259560	1259889	validated/inProgress	_	hypothetical protein
FRAAL1194	1269992	1271335	validated/inProgress	deoA	thymidine phosphorylase
FRAAL1218	1296111	1297640	validated/finished	_	hypothetical protein; putative XYPPX repeats
FRAAL1219	1298003	1298599	validated/finished	_	Transcriptional regulator TetR-family
FRAAL1256	1345820	1348627	validated/inProgress	_	hypothetical protein; putative membrane protein; putative ATP-binding region, ATPase-like domains
FRAAL1282	1379834	1380352	validated/inProgress	_	Putative merR-family transcriptional regulator
FRAAL1283	1380562	1381887	validated/inProgress	ahcY	Adenosylhomocysteinase (S-adenosyl-L-homocysteine hydrolase) (AdoHcyase)
FRAAL1295	1397391	1397840	validated/finished	_	F420-dependent oxidoreductase
FRAAL1307	1411393	1412250	validated/finished	_	Conserved protein of unknown function
FRAAL1308	1412278	1413723	validated/finished	_	Putative Peptidoglycan-binding LysM domain
FRAAL1317	1420874	1422862	validated/finished	_	hypothetical protein; putative coiled-coil domains; putative Chemotaxis protein histidine kinase domain

FRAAL1347	1454328	1455026	validated/finished	_	transcriptional regulator, tetR family
FRAAL1385	1490363	1490650	validated/finished	_	conserved hypothetical protein
FRAAL1391	1496547	1497299	validated/finished	_	conserved protein of unknown function
FRAAL1420	1521664	1522461	validated/finished	_	Nitrate/sulfonate ATP-binding cassette (ABC) transporter
FRAAL1424	1526151	1528439	validated/finished	_	Integral membrane export protein involved in sterol/hopanoid lipid transport
FRAAL1445	1548865	1549098	validated/finished	_	hypothetical protein; putative signal peptide
FRAAL1450	1553256	1554299	validated/inProgress	_	putative nitrate/sulfonate/bicarbonate ABC transporter substrate-binding protein
FRAAL1451	1554383	1555180	validated/inProgress	_	putative ABC transporter ATP-binding protein
FRAAL1452	1555177	1556091	validated/inProgress	_	putative ABC transporter, permease protein
FRAAL1453	1556184	1556990	validated/inProgress	_	putative ABC transporter permease protein
FRAAL1457	1562364	1563296	validated/finished	xdhB	Xanthine dehydrogenase, FAD binding subunit
FRAAL1458	1563554	1563994	validated/finished	xdhC	Xanthine dehydrogenase, 2Fe-2S ferredoxin-like, small subunit
FRAAL1459	1563984	1566416	validated/finished	xdhA	Xanthine dehydrogenase, large subunit; molybdenum cofactor-binding domain,
FRAAL1462	1569848	1570174	validated/finished	hiuH	Uric acid degradation bifunctional protein TTL [Includes: 2-oxo-4-hydroxy-4-carboxy-5-ureidoimidazoline decarboxylase ; 5-hydroxyisourate hydrolase]
FRAAL1474	1580216	1581598	validated/finished	trzN	dehalogenase, putative metal-dependent atrazine dechlorinase
FRAAL1507	1610195	1610572	validated/finished	_	Conserved secreted protein of unknown function
FRAAL1508	1610605	1611033	validated/inProgress	_	hypothetical protein; putative membrane protein
FRAAL1515	1616812	1617387	validated/inProgress	_	hypothetical protein; putative signal peptide
FRAAL1572	1685865	1688729	validated/inProgress	_	hypothetical protein; putative membrane protein; putative ATPase-like domain
FRAAL1614	1736409	1737476	validated/inProgress	_	hypothetical protein
FRAAL1620	1742123	1742959	validated/inProgress	_	conserved hypothetical protein; putative signal peptide
FRAAL1637	1757918	1758949	validated/inProgress	_	alcohol dehydrogenase
FRAAL1641	1764304	1765128	validated/inProgress	_	putative enoyl-CoA hydratase/isomerase
FRAAL1650	1769889	1770377	validated/inProgress	_	hypothetical protein
FRAAL1656	1773708	1774622	validated/finished	_	Enoyl-CoA hydratase
FRAAL1658	1775310	1776332	validated/finished	_	Two-component system response regulator, C-terminal effector domain of the bipartite response regulators
FRAAL1659	1776329	1777600	validated/finished	_	Two-component system regulator, GAF domain
FRAAL1660	1777623	1778819	validated/finished	_	Acyl-CoA dehydrogenase
FRAAL1661	1779104	1779652	validated/finished	_	Conserved protein of unknown function; snoAL and NTF domains
FRAAL1662	1779701	1780270	validated/finished	_	Conserved protein of unknown function; putative NTF2-like and Rhodopsin-like GPCR domains
FRAAL1663	1780372	1782030	validated/finished	lcfB	Long-chain-fatty-acid--CoA ligase
FRAAL1664	1782156	1782641	validated/finished	_	Flavin reductase domain protein, FMN-binding protein
FRAAL1665	1782694	1784289	validated/finished	aldH	Acetaldehyde dehydrogenase
FRAAL1666	1784249	1785430	validated/finished	fadE	Acyl-CoA dehydrogenase
FRAAL1667	1785443	1787200	validated/finished	accD	acetyl/propionyl CoA carboxylase, beta subunit
FRAAL1668	1787206	1789308	validated/finished	accC	Acetyl-/propionyl-coenzyme A carboxylase alpha chain [Includes: Biotin carboxylase; Biotin carboxyl carrier protein (BCCP)]
FRAAL1683	1813299	1814228	validated/inProgress	_	Putative zinc-binding oxidoreductase
FRAAL1684	1814404	1815987	validated/inProgress	_	hypothetical protein; putative IMP dehydrogenase / GMP reductase domain
FRAAL1685	1816556	1818718	validated/inProgress	_	hypothetical protein; putative IMP dehydrogenase / GMP reductase domain
FRAAL1687	1824894	1825961	validated/inProgress	_	hypothetical protein
FRAAL1691	1829447	1830628	validated/inProgress	_	Epoxide hydrolase
FRAAL1710	1846684	1847937	validated/inProgress	_	Putative ATP/GTP binding protein
FRAAL1745	1873447	1875381	validated/inProgress	_	Tellurium resistance protein terE
FRAAL1749	1877569	1879200	validated/finished	_	Aldehyde dehydrogenase
FRAAL1753	1880790	1882274	validated/inProgress	_	Putative WD-40 repeat protein
FRAAL1754	1883668	1885230	validated/inProgress	_	hypothetical protein
FRAAL1755	1885541	1886998	validated/inProgress	_	Putative transcriptional regulator
FRAAL1758	1888058	1888417	validated/inProgress	_	hypothetical protein
FRAAL1769	1897990	1898754	validated/finished	_	conserved protein of unknown function
FRAAL1784	1918745	1918990	validated/inProgress	_	hypothetical protein
FRAAL1786	1919989	1920543	validated/inProgress	_	hypothetical protein
FRAAL1791	1924844	1926721	validated/inProgress	lysS	Lysyl-tRNA synthetase (Lysine--tRNA ligase) (LysS)
FRAAL1810	1947019	1948245	validated/finished	_	Aromatic-ring hydroxylase
FRAAL1812	1948928	1949299	validated/finished	_	conserved protein of unknown function; putative Winged helix DNA-binding domain
FRAAL1813	1949296	1949745	validated/inProgress	_	hypothetical protein
FRAAL1814	1949742	1950935	validated/inProgress	_	Putative sensor-like histidine kinase
FRAAL1835	1976287	1976541	validated/inProgress	_	hypothetical protein
FRAAL1840	1981040	1981759	validated/finished	ssb	Single-strand DNA binding protein
FRAAL1886	2042441	2043811	validated/inProgress	_	Putative membrane transport protein
FRAAL1890	2046972	2048417	validated/finished	glxD	Glutamate synthase large subunit-like protein
FRAAL1891	2048420	2049667	validated/inProgress	_	putative sarcosine oxidase subunit beta
FRAAL1892	2049697	2050293	validated/inProgress	_	putative HTH-type transcriptional regulator
FRAAL1894	2051451	2053136	validated/inProgress	_	hypothetical protein
FRAAL1895	2053146	2053919	validated/inProgress	_	putative Heat shock protein 16 (16 kDa heat shock protein)
FRAAL1945	2113835	2114227	validated/inProgress	_	hypothetical protein; putative signal peptide
FRAAL1956	2123854	2124789	validated/inProgress	_	putative Quinone oxidoreductase
FRAAL1959	2126191	2127573	validated/inProgress	_	putative glycosyltransferase (N-glycosyltransferase) (N-glycosyl transferase NGT)
FRAAL1988	2156975	2157661	validated/inProgress	_	putative secreted protein
FRAAL1993	2161333	2162127	validated/inProgress	_	putative Tyrosine specific protein phosphatase

FRAAL1994	2162256	2162849	validated/inProgress	_	hypothetical protein; putative membrane protein
FRAAL2040	2213185	2214417	validated/inProgress	_	Putative glycosyl transferase
FRAAL2046	2220368	2221249	validated/inProgress	_	putative Glycosyl transferase-like protein
FRAAL2142	2338845	2339186	validated/inProgress	_	hypothetical protein
FRAAL2208	2420975	2421763	validated/inProgress	lspA	Lipoprotein signal peptidase (Prolipoprotein signal peptidase) (Signal peptidase II) (SPase II) (partial match)
FRAAL2241	2446828	2450469	validated/inProgress	_	Putative serine/threonine protein kinase
FRAAL2306	2515113	2515754	validated/inProgress	_	conserved hypothetical protein
FRAAL2311	2519979	2520923	validated/inProgress	purU	formyltetrahydrofolate hydrolase
FRAAL2338	2556204	2556824	validated/finished	_	hypothetical protein
FRAAL2345	2565031	2567202	validated/inProgress	_	hypothetical protein
FRAAL2349	2573783	2574739	validated/inProgress	_	Putative sugar kinase
FRAAL2354	2578819	2580321	validated/finished	_	Transcriptional regulator, XRE family
FRAAL2359	2586449	2587117	validated/inProgress	_	Putative tetR-family transcriptional regulator
FRAAL2362	2589788	2590942	validated/inProgress	_	Acyl-CoA dehydrogenase, long-chain specific (LCAD)
FRAAL2363	2591607	2593232	validated/inProgress	_	putative ABC transporter ATP-binding protein; putative galactose-binding domain
FRAAL2366	2594387	2596000	validated/inProgress	_	Putative transmembrane efflux protein
FRAAL2374	2600288	2601460	validated/inProgress	_	hypothetical protein
FRAAL2384	2610315	2611157	validated/inProgress	_	putative ABC-type transporter ATP-binding protein protein; part of putative ABC transporter of unknown substrate
FRAAL2388	2614407	2614790	validated/inProgress	hypA1	Hydrogenase nickel incorporation protein hypA
FRAAL2389	2614909	2615688	validated/inProgress	hypB1	hydrogenase-3 accessory protein for metallocenter assembly with P-loop containing NTP hydrolase domain
FRAAL2390	2615884	2617422	validated/inProgress	_	conserved hypothetical protein
FRAAL2391	2617419	2617943	validated/inProgress	hupD1	Hydrogenase maturation protein hupD
FRAAL2392	2617962	2619041	validated/inProgress	hupS1	uptake hydrogenase small subunit precursor (Hydrogenlyase) (Membrane-bound hydrogenase small subunit)
FRAAL2393	2619041	2620834	validated/inProgress	hupL1	Uptake hydrogenase large subunit (Hydrogenlyase) (Membrane-bound hydrogenase large subunit)
FRAAL2394	2620947	2621489	validated/finished	_	Thioredoxin-like protein
FRAAL2395	2621486	2622343	validated/finished	_	conserved protein of unknown function
FRAAL2396	2622397	2622606	validated/inProgress	_	hypothetical protein
FRAAL2404	2629554	2630336	validated/inProgress	_	conserved hypothetical protein
FRAAL2428	2649094	2649609	validated/inProgress	_	putative NADH dehydrogenase/NAD(P)H nitroreductase
FRAAL2435	2654768	2655430	validated/inProgress	_	putative acyl carrier protein phosphodiesterase 1 (ACP phosphodiesterase 1)
FRAAL2462	2683581	2684033	validated/inProgress	_	conserved hypothetical protein
FRAAL2468	2688530	2689561	validated/inProgress	_	Esterase
FRAAL2480	2698844	2700802	validated/inProgress	_	putative ABC transporter ATP-binding protein
FRAAL2481	2700795	2702645	validated/inProgress	_	putative antibiotic ABC transporter ATP-binding protein
FRAAL2504	2725615	2726265	validated/finished	atoA	acetyl-CoA:acetoacetyl-CoA transferase, beta subunit
FRAAL2513	2734026	2735231	validated/inProgress	_	Putative Acyl-CoA dehydrogenase
FRAAL2519	2739295	2740122	validated/inProgress	_	Putative short-chain dehydrogenase
FRAAL2525	2743899	2744792	validated/inProgress	_	Putative short-chain dehydrogenase
FRAAL2625	2870775	2871917	validated/inProgress	_	Acyl-CoA dehydrogenase, long-chain specific
FRAAL2634	2879472	2880542	validated/finished	_	Putative amidohydrolase (upregulated upon contact with naphtenic acid)
FRAAL2649	2900680	2901351	validated/inProgress	_	Putative transposase (partial)
FRAAL2792	3015025	3016173	validated/inProgress	acd	Butyryl-CoA dehydrogenase
FRAAL2813	3033731	3035203	validated/inProgress	_	hypothetical protein
FRAAL2837	3063663	3064883	validated/inProgress	_	hypothetical protein
FRAAL2855	3086435	3086977	validated/inProgress	_	conserved hypothetical protein
FRAAL2903	3140673	3141830	validated/inProgress	_	UDP glycosyltransferase
FRAAL2910	3147115	3148482	validated/inProgress	_	UDP-glucuronosyltransferase
FRAAL2928	3171890	3172174	validated/finished	_	Putative HNH endonuclease
FRAAL2954	3192502	3193758	validated/inProgress	_	conserved hypothetical protein
FRAAL2981	3249642	3251177	validated/inProgress	_	putative two-component sensor
FRAAL3025	3299097	3299528	validated/inProgress	gvpA	Gas vesicle protein A
FRAAL3026	3299630	3300352	validated/finished	gvpF	Gas vesicle protein F
FRAAL3028	3301047	3302054	validated/finished	_	Polyketide cyclase in gvp synton
FRAAL3029	3302167	3302649	validated/inProgress	_	conserved hypothetical protein
FRAAL3030	3302661	3303101	validated/finished	_	Transcriptional regulator, ArsR family
FRAAL3032	3303988	3305496	validated/inProgress	_	conserved hypothetical protein
FRAAL3034	3306387	3306974	validated/inProgress	_	conserved hypothetical protein; putative membrane protein
FRAAL3039	3311671	3312699	validated/inProgress	pdhA	Pyruvate dehydrogenase E1 component, alpha subunit
FRAAL3040	3312696	3313706	validated/inProgress	pdhB	Pyruvate dehydrogenase, beta subunit (Lipoamide). (pdhB-2)
FRAAL3041	3313766	3313996	validated/inProgress	_	putative Dihydrolipoamide acyltransferases
FRAAL3050	3324505	3325218	validated/inProgress	_	hypothetical protein
FRAAL3063	3336920	3338002	validated/finished	_	ABC-type branched-chain amino acid transporter
FRAAL3078	3350285	3351217	validated/inProgress	_	transposase
FRAAL3080	3352700	3353941	validated/inProgress	_	transposase
FRAAL3116	3385004	3385582	validated/inProgress	_	conserved hypothetical protein; putative oxidoreductase activity
FRAAL3141	3412771	3414414	validated/finished	_	Protein of unknown function; putative His-Me finger endonucleases domain
FRAAL3144	3416870	3418051	validated/finished	_	Acyl-CoA dehydrogenase, long-chain specific
FRAAL3148	3421985	3422821	validated/finished	scoA	succinyl-CoA:3-ketoacid-coenzyme A transferase subunit A (Succinyl CoA:3-oxoacid CoA-transferase) (OXCT A)
FRAAL3149	3422818	3423459	validated/finished	atoA	acetyl-CoA:acetoacetyl-CoA transferase, beta subunit

FRAAL3238	3521962	3522543	validated/inProgress	_	hypothetical protein
FRAAL3250	3534207	3535754	validated/inProgress	_	Putative efflux membrane protein
FRAAL3271	3560729	3561160	validated/inProgress	_	Putative regulatory protein
FRAAL3310	3595499	3595759	validated/inProgress	_	conserved hypothetical protein
FRAAL3311	3595756	3596136	validated/inProgress	_	conserved hypothetical protein
FRAAL3347	3627931	3628752	validated/finished	_	hypothetical protein
FRAAL3383	3667656	3668603	validated/inProgress	_	hypothetical protein; putative signal peptide; putative Phenylacetic acid degradation-related domain
FRAAL3387	3671344	3672951	validated/inProgress	_	Cyclohexanone monooxygenase
FRAAL3411	3695178	3696641	validated/finished	aldH	Aminobutyraldehyde dehydrogenase
FRAAL3415	3698934	3700385	validated/inProgress	_	hypothetical protein; putative HNH endonuclease domain
FRAAL3416	3700529	3701698	validated/inProgress	_	Putative Acyl-CoA dehydrogenase
FRAAL3417	3701792	3702430	validated/inProgress	_	putative Transcriptional regulator
FRAAL3422	3707664	3708590	validated/inProgress	_	putative short-chain dehydrogenase/reductase
FRAAL3448	3736763	3737938	validated/inProgress	glpQ	Glycerophosphoryl diester phosphodiesteraseglycerophosphoryl diester phosphodiesterase
FRAAL3464	3753291	3754517	validated/finished	_	Acyl-CoA dehydrogenase
FRAAL3467	3755721	3756947	validated/finished	_	Branched-chain amino acid-binding lipoprotein transmembrane
FRAAL3484	3774586	3775482	validated/finished	_	Enoyl-CoA hydratase
FRAAL3512	3805159	3806751	validated/inProgress	_	Putative fatty-acid--CoA ligase
FRAAL3514	3807920	3809206	validated/inProgress	_	putative acyl-CoA dehydrogenase
FRAAL3515	3809497	3810309	validated/inProgress	_	putative oxidoreductase, short-chain dehydrogenase/reductase family
FRAAL3539	3839288	3840040	validated/inProgress	_	putative oxidoreductase, NAD(P)-binding domain
FRAAL3553	3853255	3854721	validated/inProgress	_	Putative acyl-CoA synthetase, long- chain fatty acid:CoA ligase
FRAAL3560	3861516	3862277	validated/inProgress	_	putative 3-oxoacyl-[acyl-carrier protein] reductase
FRAAL3574	3872399	3872962	validated/inProgress	_	hypothetical protein; putative domain
FRAAL3605	3899533	3900276	validated/inProgress	_	hypothetical protein
FRAAL3610	3903567	3904298	validated/inProgress	_	Putative oxidoreductase; (Related to short-chain alcohol dehydrogenases)
FRAAL3611	3904511	3905029	validated/inProgress	_	Putative MarR-family transcriptional regulator
FRAAL3620	3915729	3916526	validated/inProgress	_	hypothetical protein; putative NAD(P)-binding Rossmann-fold domain
FRAAL3621	3916792	3917361	validated/inProgress	_	putative TetR-family transcriptional regulator
FRAAL3664	3950438	3951334	validated/inProgress	_	conserved hypothetical protein; putative nuclease domain
FRAAL3669	3954375	3954974	validated/inProgress	_	hypothetical protein
FRAAL3670	3954977	3955435	validated/inProgress	_	putative Transcriptional regulator
FRAAL3695	3974029	3974673	validated/inProgress	_	Putative secreted protein (partial)
FRAAL3703	3980982	3981830	validated/inProgress	_	conserved hypothetical protein
FRAAL3704	3981742	3982344	validated/inProgress	_	putative TetR-family transcriptional regulator
FRAAL3738	4014415	4014807	validated/inProgress	_	Putative transcriptional regulator (partial match)
FRAAL3795	4070112	4070708	validated/inProgress	_	putative TetR-family transcriptional regulator
FRAAL3799	4075323	4076087	validated/inProgress	_	Enoyl-CoA hydratase
FRAAL3800	4076683	4077897	validated/inProgress	_	hypothetical protein; putative signal peptide
FRAAL3811	4090826	4091698	validated/inProgress	_	putative ABC-type uncharacterized transport system, substrate-binding protein, membrane component
FRAAL3813	4092636	4093436	validated/inProgress	_	ABC transporter ATP-binding protein
FRAAL3822	4099716	4101056	validated/inProgress	_	putative multidrug resistance integral membrane efflux protein
FRAAL3823	4101273	4101950	validated/inProgress	_	putative TetR family transcriptional regulator
FRAAL3825	4103582	4103776	validated/inProgress	_	Putative ferredoxin (partial)
FRAAL3834	4112440	4112958	validated/finished	_	Flavin-dependent reductase, FMN-binding
FRAAL3836	4114604	4115446	validated/inProgress	_	putative O-methyltransferase involved in polyketide biosynthesis
FRAAL3851	4127005	4129416	validated/inProgress	_	putative serine/threonine protein kinase
FRAAL3853	4129974	4130591	validated/finished	_	Transcriptional regulator, TetR family
FRAAL3891	4179775	4181097	validated/inProgress	_	putative secreted protein
FRAAL3893	4181145	4182197	validated/inProgress	_	putative ABC transporter glutamine-binding protein GlnH
FRAAL3896	4186550	4187530	validated/inProgress	_	Putative protein phosphatase
FRAAL3897	4187511	4188839	validated/inProgress	_	conserved hypothetical protein
FRAAL3898	4189025	4189429	validated/inProgress	_	hypothetical protein
FRAAL3901	4193272	4195026	validated/inProgress	_	putative Phytoene dehydrogenase
FRAAL3961	4260931	4262001	validated/inProgress	_	putative Trypsin-like serine proteases
FRAAL3970	4267938	4268750	validated/inProgress	_	putative repressor
FRAAL3971	4268933	4270000	validated/inProgress	_	putative short-chain acyl dehydrogenase
FRAAL4021	4325363	4326607	validated/inProgress	_	putative Sigma factor PP2C-like phosphatases
FRAAL4022	4326796	4332276	validated/inProgress	_	Putative two component sensor serine/threonine kinase
FRAAL4067	4370645	4371502	validated/inProgress	_	conserved hypothetical protein
FRAAL4087	4422039	4422824	validated/inProgress	_	putative dipeptide transport protein (ABC superfamily, membrane)
FRAAL4088	4422958	4423875	validated/inProgress	_	ABC transporter permease
FRAAL4126	4457663	4458835	validated/inProgress	_	hypothetical protein; putative membrane protein
FRAAL4180	4542761	4543153	validated/inProgress	_	conserved hypothetical protein
FRAAL4187	4548805	4550193	validated/inProgress	_	hypothetical protein
FRAAL4221	4586965	4588062	validated/inProgress	_	conserved hypothetical protein
FRAAL4222	4588626	4589168	validated/inProgress	_	conserved hypothetical protein; putative signal peptide
FRAAL4239	4601176	4601829	validated/finished	_	Putative Rho termination factor, N-terminal domain
FRAAL4242	4604749	4605792	validated/inProgress	_	hypothetical protein

FRAAL4259	4623650	4624054	validated/inProgress	_	Putative HTH-type transcriptional regulator
FRAAL4291	4650565	4650885	validated/inProgress	_	hypothetical protein
FRAAL4294	4653546	4654313	validated/inProgress	_	hypothetical protein; putative signal peptide
FRAAL4320	4685412	4686725	validated/inProgress	_	putative hydroxylase
FRAAL4340	4708356	4709603	validated/finished	_	hypothetical protein; putative membrane protein
FRAAL4361	4733618	4735462	validated/inProgress	_	Conserved hypothetical protein
FRAAL4364	4738725	4739636	validated/inProgress	_	putative RpiR familytranscriptional regulator with phosphosugar-binding domain
FRAAL4404	4786199	4786375	validated/inProgress	_	hypothetical protein
FRAAL4421	4808582	4809514	validated/inProgress	_	putative FMN reductase
FRAAL4422	4809770	4810663	validated/inProgress	_	putative Coenzyme F420-dependent reductase
FRAAL4430	4818938	4819291	validated/finished	_	Putative surface carbohydrate-binding protein
FRAAL4437	4824109	4824558	validated/inProgress	_	hypothetical protein
FRAAL4441	4827755	4828603	validated/inProgress	_	putative lysR-type transcriptional regulator
FRAAL4446	4832058	4833722	validated/inProgress	_	putative ABC transporter dipeptide/oligopeptide binding protein
FRAAL4447	4833719	4834807	validated/inProgress	_	Putative ABC-type dipeptide/oligopeptide transport system, permease component
FRAAL4448	4834939	4835730	validated/inProgress	_	Putative dipeptide/oligopeptide ABC transporter permease
FRAAL4498	4884005	4884364	validated/inProgress	_	hypothetical protein
FRAAL4531	4919186	4920340	validated/inProgress	_	putative transcription regulator
FRAAL4539	4928433	4930289	validated/inProgress	_	Putative ABC-type transport system, ATPase and permease components
FRAAL4540	4930448	4931344	validated/finished	_	Enoyl-CoA hydratase/isomerase
FRAAL4710	5097449	5098309	validated/inProgress	_	Putative oxidoreductase, short-chain alcohol dehydrogenase family
FRAAL4713	5100184	5101143	validated/finished	_	Putative enoyl-CoA hydratase/isomerase
FRAAL4715	5101895	5102488	validated/inProgress	_	Putative transcriptional regulator of the TetR family
FRAAL4716	5102637	5103398	validated/inProgress	_	Putative oxidoreductase
FRAAL4721	5107346	5108296	validated/inProgress	_	hypothetical protein; putative signal peptide
FRAAL4722	5108466	5110484	validated/finished	_	putative ATP-binding component of an ABC transport protein
FRAAL4728	5120334	5120582	validated/inProgress	_	hypothetical protein
FRAAL4730	5121133	5122752	validated/finished	_	Putative permease of the major facilitator superfamily
FRAAL4747	5139365	5140288	validated/inProgress	_	Putative stress-inducible protein; putative adenine nucleotide-binding domain
FRAAL4749	5142847	5144160	validated/inProgress	_	putative membrane protein
FRAAL4751	5144435	5144983	validated/inProgress	_	putative tetR family transcriptional regulator
FRAAL4755	5147703	5148992	validated/inProgress	_	putative membrane phosphatase
FRAAL4764	5157858	5160299	validated/inProgress	_	conserved hypothetical protein; putative Formyl-CoA transferase
FRAAL4765	5160385	5162766	validated/inProgress	_	Putative enoyl-CoA hydratase
FRAAL4766	5162773	5163621	validated/inProgress	_	Putative enoyl-CoA hydratase/isomerase
FRAAL4767	5163740	5164579	validated/inProgress	_	putative acyl-CoA thioesterase
FRAAL4781	5178512	5179087	validated/finished	_	Acyl-coenzyme A thioesterase
FRAAL4782	5179208	5179780	validated/finished	_	Putative acyl-coenzyme A thioesterase
FRAAL4791	5189285	5190373	validated/inProgress	_	Putative monooxigenase.
FRAAL4797	5196418	5197452	validated/inProgress	_	Putative dipeptide transport protein of the ABC superfamily, membrane component
FRAAL4798	5197595	5199163	validated/inProgress	_	Putative dipeptide ABC transporter precursor, membrane component
FRAAL4802	5202157	5203092	validated/inProgress	_	putative monooxygenase
FRAAL4803	5203291	5203914	validated/inProgress	_	Putative TetR family transcriptional regulator
FRAAL4830	5226751	5227884	validated/inProgress	ssuD	alkanesulfonate monooxygenase, FMNH(2)-dependent
FRAAL4832	5229094	5230455	validated/inProgress	_	putative monooxygenase
FRAAL4833	5230452	5231954	validated/inProgress	_	Putative monooxygenase
FRAAL4837	5234934	5235986	validated/inProgress	dppC	ABC-type dipeptide/oligopeptide/nickel transport systems, permease component
FRAAL4838	5235983	5236933	validated/inProgress	dppB	ABC-type dipeptide/oligopeptide/nickel transport systems, permease component
FRAAL4839	5236980	5238500	validated/finished	_	ABC peptide transporter; membrane component
FRAAL4841	5239217	5240326	validated/inProgress	_	putative oxygenase
FRAAL4847	5245909	5246877	validated/inProgress	_	putative zinc-binding NADP-dependent dehydrogenase
FRAAL4852	5251721	5252518	validated/inProgress	_	Putative transcriptional regulator (GntR-family)
FRAAL4879	5280485	5281630	validated/finished	_	amidohydrolase
FRAAL4895	5296328	5296780	validated/finished	_	putative cation transporter regulator
FRAAL4909	5320755	5321405	validated/inProgress	_	MutT/nudix family protein
FRAAL4911	5322309	5323076	validated/inProgress	_	Chitooligosaccharide deacetylase (Nodulation protein B homolog) (partial match)
FRAAL4930	5342018	5343205	validated/inProgress	_	conserved hypothetical protein; putative monooxygenase
FRAAL4941	5352674	5353468	validated/inProgress	_	putative Guanidinoacetate N-methyltransferase
FRAAL4959	5380087	5380935	validated/finished	_	Putative tryptophan-associated membrane protein
FRAAL5000	5428413	5428982	validated/inProgress	_	hypothetical protein
FRAAL5013	5441040	5442581	validated/inProgress	_	hypothetical protein
FRAAL5020	5454412	5454918	validated/inProgress	_	hypothetical protein
FRAAL5030	5461276	5461773	validated/inProgress	_	hypothetical protein; putative signal peptide
FRAAL5032	5461899	5462939	validated/inProgress	_	hypothetical protein; putative signal peptide
FRAAL5033	5462999	5464180	validated/finished	_	Secreted subtilisin-like serine protease
FRAAL5041	5473499	5474710	validated/inProgress	_	putative HTH-type transcriptional regulator
FRAAL5158	5591916	5592224	validated/finished	_	Protein of unknown function
FRAAL5185	5621241	5621579	validated/inProgress	_	conserved hypothetical protein
FRAAL5217	5654405	5655577	validated/inProgress	_	hypothetical protein

FRAAL5285	5730463	5731536	validated/finished	_	Putative amidohydrolase
FRAAL5341	5798506	5800242	validated/finished	_	putative oxidoreductase
FRAAL5351	5806376	5808145	validated/inProgress	_	Putative Na+/H+ antiporter; putative membrane protein
FRAAL5352	5808100	5809557	validated/inProgress	_	Putative regulatory protein
FRAAL5354	5810702	5811550	validated/inProgress	_	hypothetical protein
FRAAL5360	5814786	5815844	validated/inProgress	_	hypothetical protein; putative signal peptide
FRAAL5361	5815795	5816559	validated/inProgress	_	hypothetical protein; putative sortase domain
FRAAL5387	5839347	5841125	validated/inProgress	_	putative ATP-binding ABC transporter protein
FRAAL5388	5841206	5842114	validated/inProgress	_	putative dipeptide transport protein (ABC superfamily, membrane)
FRAAL5389	5842118	5843140	validated/inProgress	_	putative ABC transporter permease protein
FRAAL5415	5871724	5872071	validated/inProgress	_	hypothetical protein; putative carboxymuconolactone decarboxylase domain
FRAAL5476	5921166	5922083	validated/inProgress	_	conserved hypothetical protein
FRAAL5483	5930092	5930880	validated/finished	echA	Enoyl-CoA hydratase
FRAAL5515	5963181	5964449	validated/inProgress	_	Putative lipoprotein
FRAAL5531	5980024	5980212	validated/inProgress	_	hypothetical protein
FRAAL5536	5983711	5984220	validated/inProgress	_	hypothetical protein
FRAAL5540	5986427	5986906	validated/inProgress	_	Conserved hypothetical protein
FRAAL5553	6003814	6004155	validated/inProgress	_	hypothetical protein; putative signal peptide
FRAAL5568	6019001	6019663	validated/inProgress	_	Antibiotic resistance protein
FRAAL5572	6022912	6025518	validated/finished	_	Putative transcriptional regulator
FRAAL5600	6045711	6046568	validated/inProgress	_	putative DNA-binding protein
FRAAL5605	6049437	6050432	validated/inProgress	_	Transcriptional regulator (AraC-family)
FRAAL5607	6051507	6051722	validated/inProgress	_	Hypothetical protein
FRAAL5610	6053610	6056123	validated/inProgress	_	hypothetical protein
FRAAL5611	6056179	6057525	validated/inProgress	_	hypothetical protein
FRAAL5628	6078867	6080012	validated/inProgress	_	Hypothetical protein; putative TolB protein precursor
FRAAL5629	6080056	6080685	validated/inProgress	_	putative Redox-sensitive soxR-family transcriptional activator
FRAAL5650	6105098	6107560	validated/inProgress	_	conserved hypothetical protein; putative membrane protein
FRAAL5663	6124118	6125008	validated/inProgress	_	hypothetical protein; putative Acetyltransferases (isoleucine patch superfamily)
FRAAL5683	6144570	6146495	validated/finished	_	Putative DNA-binding protein
FRAAL5700	6173245	6173919	validated/inProgress	_	Putative regulatory protein nrdR
FRAAL5761	6246528	6246719	validated/inProgress	_	hypothetical protein
FRAAL5800	6286271	6287068	validated/finished	_	hypothetical protein; putative aromatic acid decarboxylase
FRAAL5848	6332472	6334064	validated/inProgress	serA	D-3-phosphoglycerate dehydrogenase (PGDH)
FRAAL5875	6364641	6365477	validated/finished	_	Putative oxidoreductase
FRAAL5908	6412080	6412550	validated/finished	_	conserved protein of unknown function
FRAAL5923	6427160	6427525	validated/finished	_	Anti-sigma factor antagonist
FRAAL5928	6430643	6430888	validated/inProgress	_	hypothetical protein
FRAAL5929	6430915	6431226	validated/inProgress	_	Hypothetical protein
FRAAL6013	6517875	6519314	validated/finished	amiB	Amidase, Asp-tRNAAsn/Glu-tRNAGln amidotransferase A subunit
FRAAL6021	6524424	6525776	validated/finished	_	Putative hydrolase
FRAAL6062	6576617	6578074	validated/finished	_	putative membrane protein involved in divalent ion export
FRAAL6063	6578071	6579114	validated/finished	_	putative membrane protein involved in export
FRAAL6065	6579682	6580422	validated/finished	_	Putative monooxygenase
FRAAL6137	6660392	6661453	validated/inProgress	_	Putative ribosylglycoyhydrolase
FRAAL6157	6681595	6682290	validated/inProgress	_	conserved hypothetical protein; putative Pantothenate kinase
FRAAL6210	6735920	6739129	validated/inProgress	leuS	Leucyl-tRNA synthetase (Leucine--tRNA ligase)
FRAAL6229	6757436	6758260	validated/inProgress	udgB	Uracil-DNA glycosylase
FRAAL6268	6807173	6807766	validated/inProgress	_	putative membrane protein
FRAAL6270	6807763	6808506	validated/inProgress	_	hypothetical protein; putative signal peptide
FRAAL6271	6808706	6809137	validated/inProgress	_	hypothetical protein
FRAAL6272	6809134	6809322	validated/inProgress	_	conserved hypothetical protein; putative membrane protein
FRAAL6275	6810505	6810918	validated/inProgress	_	conserved hypothetical protein
FRAAL6306	6844867	6847425	validated/inProgress	_	hypothetical protein; putative Putative serine/threonine protein kinase domain
FRAAL6309	6849590	6850570	validated/inProgress	_	putative oxidoreductase
FRAAL6316	6857704	6858300	validated/inProgress	_	hypothetical protein
FRAAL6386	6940019	6940630	validated/inProgress	_	putative two-component system response regulator
FRAAL6387	6940686	6941933	validated/inProgress	_	putative two-component system sensor kinase
FRAAL6389	6942090	6942317	validated/inProgress	_	conserved hypothetical protein; Putative membrane protein (partial)
FRAAL6402	6952795	6953127	validated/inProgress	_	hypothetical protein
FRAAL6411	6960410	6963772	validated/inProgress	_	hypothetical protein; putative serine/threonine-protein kinase
FRAAL6428	6989158	6989946	validated/inProgress	_	Transcriptional regulator (HTH-type)
FRAAL6437	7001501	7004293	validated/finished	_	Putative membrane-bound cysteine protease with a transglutaminase domain
FRAAL6453	7017233	7018375	validated/inProgress	_	Oxidoreductase, electron transfer component
FRAAL6454	7018372	7019517	validated/inProgress	desA	Linoleoyl-CoA desaturase (DELTA(6)-desaturase)
FRAAL6492	7060719	7061090	validated/inProgress	_	hypothetical protein
FRAAL6493	7061153	7061851	validated/inProgress	_	hypothetical protein
FRAAL6497	7063832	7064941	validated/inProgress	_	conserved hypothetical protein
FRAAL6501	7069164	7069778	validated/inProgress	_	hypothetical protein; putative signal peptide

FRAAL6511	7083945	7084799	validated/inProgress	_	Hypothetical protein
FRAAL6516	7088393	7090576	validated/inProgress	_	hypothetical protein; putative membrane protein
FRAAL6553	7129736	7130620	validated/inProgress	_	conserved hypothetical protein
FRAAL6565	7144614	7145810	validated/inProgress	_	Hypothetical protein; Putative septum site determining protein
FRAAL6568	7148328	7148579	validated/inProgress	_	conserved hypothetical protein
FRAAL6572	7150072	7150932	validated/inProgress	_	hypothetical protein
FRAAL6653	7250627	7251421	validated/finished	_	Secreted CAP protein
FRAAL6694	7299935	7300315	validated/finished	_	conserved protein of unknown function
FRAAL6705	7314692	7315150	validated/inProgress	_	hypothetical protein
FRAAL6715	7323709	7324662	validated/inProgress	_	Hypothetical protein; putative Protein kinase-like domain
FRAAL6717	7326356	7328869	validated/inProgress	_	conserved hypothetical protein; putative signal peptide
FRAAL6724	7333518	7334219	validated/finished	purQ	Phosphoribosylformylglycinamidine synthase I (FGAM synthase I)
FRAAL6730	7338262	7340649	validated/finished	purL	Phosphoribosylformylglycinamidine synthase II (FGAM synthase II)
FRAAL6738	7347692	7347973	validated/inProgress	purS	phosphoribosylformylglycinamidine synthase
FRAAL6746	7352718	7353353	validated/inProgress	_	putative TetR family transcriptional regulator
FRAAL6769	7375790	7377367	validated/finished	_	Conserved protein of unknown function, putative coiled-coil domains
FRAAL6773	7379912	7381807	validated/inProgress	_	hypothetical protein
FRAAL6791	7398897	7399586	validated/inProgress	_	hypothetical protein; Putative methyltransferase
FRAAL6794	7401024	7401473	validated/inProgress	_	putative transcriptional regulatory protein
FRAAL6795	7401542	7402399	validated/inProgress	qorB	Quinone oxidoreductase
FRAAL6820	7425925	7426512	validated/inProgress	_	putative methyltransferase
FRAAL6854	7456168	7456431	validated/inProgress	_	hypothetical protein
FRAL4387	4780796	4781107	validated/inProgress	_	hypothetical protein
FRAL6231	6787064	6787216	validated/inProgress	_	hypothetical protein

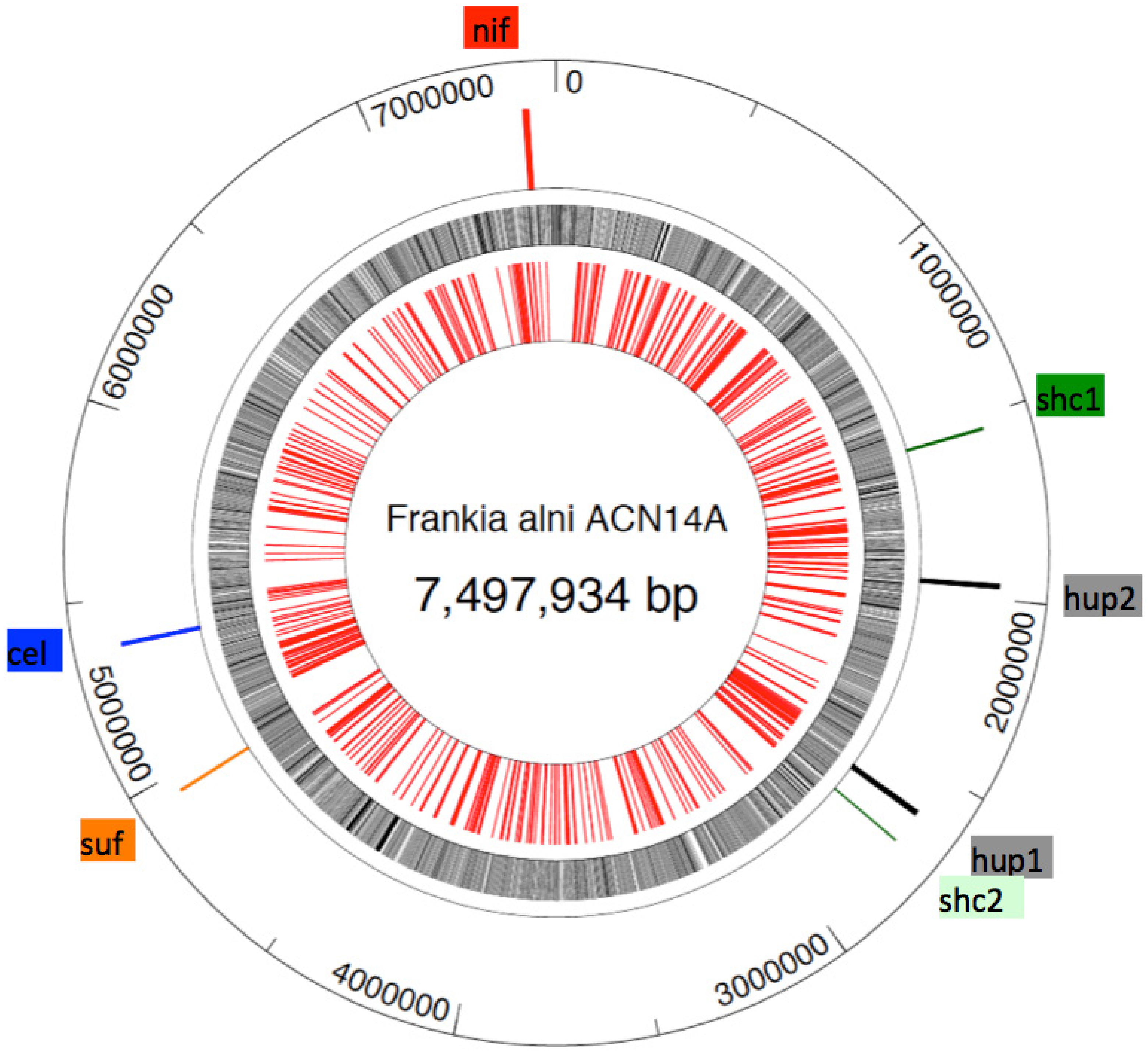

Supplementary Figure S1. Circular representation of the *Frankia alni* genome with from the inside out (in red) the genes absent from Ag45/Mut15 and AgPM24 at a threshold of 50% AA, all genes present (in gray) in ACN14a, and finally the known symbiosis genes with clockwise from the top the hopene synthesis and SHC genes cluster (SHC1 in dark green), the hydrogenase uptake #2 hup cluster (upregulated in symbiosis) in gray, the hydrogenase uptake #1 (upregulated in free-living condition) in gray, the SHC#2 gene in light green, the iron-sulfur cluster (suf in orange), the cellulase/cellulose synthase

cluster (cel in blue) and the nitrogenase cluster (nif in red).
